# Exploring the Anti-Acne Potential of Impepho [*Helichrysum odoratissimum* (L.) Sweet] to Combat *Cutibacterium acnes* Virulence

**DOI:** 10.3389/fphar.2019.01559

**Published:** 2020-01-30

**Authors:** Marco Nuno De Canha, Slavko Komarnytsky, Lenka Langhansova, Namrita Lall

**Affiliations:** ^1^ Department of Plant and Soil Sciences, University of Pretoria, Pretoria, South Africa; ^2^ Department of Food, Bioprocessing and Nutrition Sciences, Plants for Human Health Institute, North Carolina State University, Kannapolis, NC, United States; ^3^ Laboratory of Plant Biotechnologies, Institute of Experimental Botany, The Czech Academy of Sciences, Prague, Czechia; ^4^ School of Natural Resources, University of Missouri, Columbia, MO, United States; ^5^ College of Pharmacy, JSS Academy of Higher Education and Research, Mysuru, India

**Keywords:** *Helichrysum odoratissimum* (L.) Sweet, *Cutibacterium acnes*, anti-acne, antibiofilm, pathogenesis

## Abstract

The Gram-positive bacterium *Cutibacterium acnes* (previously *Propionibacterium acnes*), plays an important role in the pathogenesis and progression of the dermatological skin disorder acne vulgaris. The methanolic extract of *Helichrysum odoratissimum* (L.) Sweet (HO-MeOH) was investigated for its ability to target bacterial growth and pathogenic virulence factors associated with acne progression. The gas chromatography–mass spectrometry (GC-MS) analysis of HO-MeOH identified *α*-humulene (3.94%), *α*-curcumene (3.74%), and caryophyllene (8.12%) as major constituents, which correlated with previous reports of other *Helichrysum* species. The HO-MeOH extract exhibited potent antimicrobial activity against *C. acnes* (ATCC 6919) with a minimum inhibitory concentration (MIC) of 7.81 µg/ml. It enhanced the antimicrobial activity of benzoyl peroxide (BPO). The extract showed high specificity against *C*. *acnes* cell aggregation at sub-inhibitory concentrations, preventing biofilm formation. Mature *C*. *acnes* biofilms were disrupted at a sub-inhibitory concentration of 3.91 µg/ml. At 100 µg/ml, HO-MeOH reduced interleukin-1α (IL-1α) cytokine levels in *C*. *acnes*-induced human keratinocytes (HaCaT) by 11.08%, highlighting its potential as a comedolytic agent for the treatment of comedonal acne. The extract exhibited a 50% inhibitory concentration (IC_50_) of 157.50 µg/ml against lipase enzyme activity, an enzyme responsible for sebum degradation, ultimately causing inflammation. The extract’s anti-inflammatory activity was tested against various targets associated with inflammatory activation by the bacterium. The extract inhibited pro-inflammatory cytokine levels of IL-8 by 48.31% when compared to *C*. *acnes*-induced HaCaT cells at 7.81 µg/ml. It exhibited cyclooxygenase-II (COX-II) enzyme inhibition with an IC_50_ of 22.87 µg/ml. Intracellular nitric oxide (NO) was inhibited by 40.39% at 7.81 µg/ml when compared with NO production in lipopolysaccharide (LPS)-induced RAW264.7 cells. The intracellular NO inhibition was potentially due to the 2.14 fold reduction of inducible nitric oxide synthase (iNOS) gene expression. The HO-MeOH extract exhibited an IC_50_ of 145.45 µg/ml against virulent hyaluronidase enzyme activity, which is responsible for hyaluronan degradation and scar formation. This study provides scientific validation for the traditional use of *H. odoratissimum* as an ointment for pimples, not only due to its ability to control *C*. *acnes* proliferation but also due to its inhibitory activity on various targets associated with bacterial virulence leading to acne progression.

## Introduction

Acne vulgaris (AV) is a chronic inflammatory skin disorder of the pilosebaceous, localized on the face, chest, and back. This skin condition affects approximately 85% of people but is particularly common among adolescents. The disorder is characterized by the formation of non-inflammatory comedone lesions, more commonly referred to as blackheads or whiteheads. In more severe cases, the formation of inflammatory papules, pustules, nodules, and cysts is common. Acne vulgaris persistence and unresponsiveness to antibiotic treatments can result in the formation of atrophic (sunken) and hypertrophic scars. The formation of lesions and scars on visible areas of the skin not only affect aesthetic beauty but contribute to psychological problems affecting self-confidence and self-esteem. The progression of AV arises from four main events, including abnormal keratinocyte proliferation, increases in sebum production, increased *Cutibacterium acnes* proliferation, and activation of inflammatory cascades. The Gram-positive rod, *C. acnes* is of particular importance in acne progression. This bacterium contributes to disease progression with its ability to modulate keratinocyte proliferation, secrete virulent enzymes involved in sebum degradation (lipase) and tissue injury (hyaluronidase) and activating skin innate immunity through the activation of keratinocytes, sebocytes, and peripheral blood mononuclear cells resulting in the production of pro-inflammatory cytokines interleukin-1β (IL-1β), IL-6, IL-8, IL-12, IL-17, TNF-α, and GM-CSF (granulocyte-macrophage colony-stimulating factor) (Dessinioti and Katsambas, 2017; Jeong and Kim, 2017; Han et al., 2018). The biofilm growth form of *C. acnes* is a major contributor to antibiotic resistance and pathogenesis, with biofilm-forming strains of the bacterium being associated with more severe AV (Coenye et al., 2008). The genome sequence of *C. acnes* has provided substantial evidence with regards to the presence of genes that contribute to the ability of this microorganism to form biofilms. In the early stages of biofilm development, the attachment of bacterial cells is an important step preceding the maturation of the biofilm structure. Gene clusters coding for the formation of polysaccharide capsule biosynthesis made up of glycocalyx polymers are said to contribute to *C. acnes* adhesion to surfaces (Burkhart and Burkhart, 2007). The attachment of *C. acnes* is not only limited to structures found on the skin, but this growth form has also been identified on orthopedic bone implants made from polymethylmethacrylate, titanium alloys, silicone, and even steel indicating the adaptive adhesion ability of this microorganism (Ramage et al., 2003; Achermann et al., 2014). Abnormal keratinocyte proliferation plays a crucial role in the pathogenesis of *C. acnes*. Hyper-keratinization results in an imbalance of the shedding to proliferating keratinocyte ratio, clogging the pilosebaceous follicle, ultimately resulting in the formation of micro-comedones (De Medeiros-Ribeiro et al., 2015). The cytokine interleukin-1α (IL-1α) has been detected in both diseased and healthy epidermis in the keratinocytes (Walters et al., 1995). *C. acnes* is known to possess a glycerol-ester hydrolase A (GehA) lipase enzyme involved in the degradation of sebum triacylglycerides resulting in the release of glycerol and free fatty acids. Glycerol is used as a nutrient source for the *C. acnes* bacterium, and the free fatty acids arrange themselves between keratinocytes, increasing bacterial cell adhesion, and enhancing biofilm formation within the pilosebaceous unit (Falcocchio et al., 2006). It is, therefore, an important target to consider when testing extracts or compounds for anti-acne activity. Sebocytes are specialized cells forming part of the pilosebaceous unit. These cells are responsible for the production of lipid droplets, functioning as a moisturizer for the skin. They are also immunocompetent cells contributing to immune responses in the skin, including the production of cytokines and other inflammatory mediators. Alongside their contribution to skin barrier function, keratinocytes also form part of many pathophysiological processes acting as a bridge between the external environment and the host. Keratinocytes elicit and maintain the skin immune response through the secretion of soluble factors, including cytokines, as well as antimicrobial peptides (Nagy et al., 2005). Sebocytes in follicles colonized with *C. acnes* have shown increased cyclooxygenase-II (COX-II) expression (Makrantonaki et al., 2011; Mattii et al., 2018). The production of excess PGE_2_ results in sebaceous gland enlargement and increased sebum production, favoring *C. acnes* proliferation (Ottaviani et al., 2010). *C. acnes* results in the production of nitric oxide (NO) through chemotaxis and activation of neutrophil cells. These increased levels of NO production within the pilosebaceous follicles causes irritation and rupture of the follicular wall, ultimately leading to the formation of inflammatory lesions (Portugal et al., 2007). Hyaluronic acid (HA) is a glycosaminoglycan molecule made up of repeating disaccharide units of *β*-1,4-glucuronic acid and 1,3-*N*-acetylglucosamine connected *via* a *β*-linkage. Approximately 56% of the body’s hyaluronic acid resides in the skin, with high abundance of this extracellular polysaccharide being found in the epidermis and the dermis. This polysaccharide is produced by epidermal keratinocytes and dermal fibroblasts. Bacterial hyaluronidases (HYALs) are distributed widely among Gram-positive microorganisms including *Cutibacterium* species. Hyaluronidases act by completely degrading HA into disaccharides or by degradation into a mixture of unsaturated oligosaccharides. These enzymes contribute to bacterial virulence through tissue injury, facilitating bacterial spread to deeper tissues (Kumar et al., 2016; Nazipi et al., 2017). The inhibition of hyaluronidase activity, therefore, provides an important target for scar prevention and bacterial spread.

*Helichrysum odoratissimum* (L.) Sweet is a perennial shrub of the genus *Helichrysum,* consisting of approximately 500–600 species, of which approximately 244–250 are found in South Africa (Lourens et al., 2008). The vernacular name “Impepho” is common among species of this genus and are commonly used medicinal plants. This species is well distributed in South Africa and neighboring African countries, including Lesotho, Swaziland, Mozambique, and Zimbabwe (Swelankomo, 2004). This species has a plethora of traditional uses in the treatment of wounds, burns, eczema, and as an ointment for pimples (Hutchings et al., 1996; Akaberi et al., 2019). *H. odoratissimum* is among one of the most popular species within the *Helichrysum* genus and has been traditionally used as an application for pimples and to treat other skin dermatoses, however, it was yet to be tested for its antimicrobial activity against *C. acnes* (Hutchings et al., 1996; Lourens et al., 2011; Mabona and Van Vuuren, 2013).

This study aimed to investigate the potential of *H. odoratissimum* for its ability to treat acne by directly targeting *C. acnes* growth and indirectly by targeting the virulence factors involved in the progression of AV as a result of bacterial activity, therefore, providing scientific evidence to support traditional uses.

## Materials and Methods

### Materials

#### Chemical Reagents

Tetracycline, erythromycin, benzoyl peroxide, crystal violet, actinomycin D, dimethyl sulfoxide (DMSO), Tris, KCl, 5,5’-dithio-bis-(2-nitrobenzoic acid) (DTNB), DMPTB, Triton X-100, ibuprofen, porcine hematin, L-epinephrine, formic acid, Na_2_EDTA, Griess reagent, citric acid, Na_2_HPO_4_, NaCl, BSA, NaOH, and CTAB were purchased from Merck SA (Pty) Ltd. (Sandton, Johannesburg, SA). PrestoBlue was purchased from Thermo Fisher Scientific (Randburg, South Africa). TRIzol and molecular grade water were purchased from Thermo Fisher Scientific (Waltham, Massachusetts, USA). Dexamethasone was purchased from Sigma Chemicals Co. (St. Louis, Missouri, USA). Ultra pure liquid chromatography water and acetonitrile (Romil-UpS^™^) were purchased from Microsep, South Africa. Formic acid (99% purity) was purchased from Thermo Scientific, South Africa. Fluka^®^ Analytical Ammonium hydroxide ≥ 25% in H_2_O, eluent additive for liquid chromatography (LC) was purchased from Sigma-Aldrich, South Africa.

#### Microorganisms and Cell Culture Reagents

The *C. acnes* strain (ATCC 6919), brain heart infusion (BHI) agar, and BHI broth were purchased from Anatech Instruments (Pty) Ltd (Randburg, South Africa). Human keratinocytes (HaCaT) were donated by Dr. Lester Davids (Department of Human Biology, University of Cape Town). Murine macrophages (RAW264.7), glucose, and lipopolysaccharide (LPS) (*Escherichia coli* 0127: B8) were purchased from Merck SA (Pty) Ltd. (Sandton, Johannesburg, SA). DMEM, trypsin, PBS, FBS, penicillin-streptomycin, and amphotericin B (Fungizone) were purchased from Thermo Fisher Scientific ZA (Johannesburg, RSA). TrypLE was purchased from Thermo Fisher Scientific (Waltham, Massachusetts, USA).

#### Enzymes and Kits

Human recombinant COX-II, hyaluronidase from *Streptococcus pyogenes*, and lipase from *Candida rugosa* were all purchased from Merck SA (Pty) Ltd. (Sandton, Johannesburg, SA). The XTT cell proliferation kit II was purchased from Merck SA (Pty) Ltd. (Sandton, Johannesburg, SA). The IL-1α and PGE_2_ ELISA kits were purchased from Biocom Africa (Pty) Ltd (Centurion, Pretoria, RSA). The CBA Human Inflammatory Cytokine kit was purchased from BD Biosciences (Woodmead, Pretoria, RSA). The Promega Griess Reagent System was purchased from Promega Corporation (Madison, Wisconsin, USA). The complementary DNA (cDNA) reverse transcriptase kit and the SYBR Green PCR Master Mix kits were purchased from Thermo Fisher Scientific (Waltham, Massachusetts, USA). Primers were purchased from Integrated DNA Technologies (Skokie, Illinois, USA).

### Methods

#### Plant Extraction and Identification

*H. odoratissimum* L. Sweet was collected in the December from Durban (KwaZulu, South Africa) global positioning system (GPS) coordinates: grid 2931CC 29°00’S, 31°00’E. Taxonomical species identification was performed by Ms. Magda Nel from the H. G. W. J. Schweickerdt Herbarium at the University of Pretoria, and a voucher specimen number was deposited (PRU 118963). Shade-dried plant material (leaves and stems) was ground to a fine powder. Plant material (100 g) was extracted with methanol (1:5 w/v) for 72 h with constant agitation on a shaker. The maceration was filtered through a Buchner funnel (Whatman 1.0 filter). The filtrate was then concentrated under vacuum using a Rotary evaporator (Buchi-R-200). The crude extract was then stored at 4°C until use.

#### Extract Characterization Using Gas Chromatography–Mass Spectrometry and Liquid Chromatography–Mass Spectrometry

The gas chromatography–mass spectrometry (GC-MS) analysis of methanolic extract of *H. odoratissimum* (L.) Sweet (HO-MeOH) (1 mg/ml, 0.22 µm filtered) was performed using a LECO Pegasus 4D GC-TOFMS (LECO Africa (Pty) Ltd., Kempton Park, South Africa) equipped with a GC Rxi-5SilMS (30 m, 0.25 mm ID, 0.2 µm film thickness) column (Restek, Bellefonte, PA, USA). The temperature of the injector and the interface were kept at 250°C. The following temperature program was used for analysis: 40°C (hold for 3 min) at 10°C/min to 300°C (hold for 5 min). Helium was used as a carrier gas at a constant flow rate of 1 ml.min^−1^. Splitless injection with a 30 s splitless time was used. The first 5 min of the analysis was considered as solvent delay and omitted from the final GC-MS chromatograms. The MS transfer line temperature was set at 280°C. The mass spectrometer was operated in electron impact ionization mode (EI +) at 70 eV. Detector voltage was set to 1,750 V. The scan range was set at 40–550 Da with a data acquisition rate of 10 spectra/s. Peaks were identified by mass matching to the NIST05 database.

Compound separation and peak detection of HO-MeOH using LC-MS was performed using the Waters^®^ Synapt G2 high definition mass spectrometry (HDMS) system (Waters Inc., Milford, Massachusetts, USA). The system includes a Waters Acquity Ultra Performance Liquid Chromatography (UPLC^®^) system linked with a quadrupole-time-of-flight (QTOF) instrument. MassLynx^™^ (version 4.1) software (Waters Inc., Milford, Massachusetts, USA) was used for data acquisition and processing. The internal lock mass control standard was a 2 pg/μl solution of leucine enkephalin (m/z 555.2693). Sodium formate clusters and Intellistart functionality was used to calibrate the instrument (mass range 112.936–1.132.688 Da). Resolution of 20,000 at m/z 200 [full width at half maximum (FWHM)] and mass error within 0.4 mDa were obtained.

The HO-MeOH extracts was prepared to a 1 mg/ml stock concentration and filtered using a 0.2 µm syringe filter to remove any particulates. A Kinetex^®^ 1.7 µm EVO C18 100 Å (2.1 mm ID x 100 mm length) column was used for separation. A volume of 5 µl was injected into the system using an autosampler. A reverse phase step gradient elution scheme from 97% H_2_O (0.1% formic acid) to 100% acetonitrile (0.1% formic acid) was used as the mobile phase. The gradient started with an isocratic flow (hold 0.1 min) followed by a linear increase to 100% ACN; subsequently the column was washed for 1 min followed by conditioning and re-establishing of initial conditions to allow for equilibration before the start of the next run for the complete elution scheme. The column temperature was kept constant at 40°C and the flow rate was set at 0.4 ml/min for the each run, giving a total run time of 20 min. The positive and negative ion mass spectra were collected in separate chromatographic runs (employing the same separation conditions). Mass spectral scans were collected every 0.3 s. The raw data was collected in the form of a continuous profile. Mass to charge ratios (m/z) between 50 and 1,200 Da were recorded. Quantitative data-independent acquisition (DIA) was done using two simultaneous acquisition functions with low and high collision energy (MSE approach) with a QTOF instrument. The fragmentation energy was set at 1 and 4 V (positive mode) and 1.8 and 6 V (negative mode) for the trap and collision energies, respectively. The ramping was set from 3 to 4 V and 20 to 30 V for both modes for the trap and transfer collision energy, respectively. The electrospray ionization (ESI) mode conditions were set as follows; capillary voltage for ESI was 2.6 and 2.0 kV for positive and negative mode ionization, respectively. The source temperature for both modes was set at 120°C. the sampling cone voltage at 25 V for negative mode and 40 V for positive mode. The extraction cone voltage was set at 4.0 V for both modes. Cone gas (nitrogen) flow was set at 10.0 L/Hr for both modes. The desolvation temperature was set at 300°C with a gas (nitrogen) flow of 600.0 L/h.

#### Determination of Minimum Inhibitory Concentration

Susceptibility of the *C. acnes* (ATCC 6919) strain was tested using the microdilution broth assay as described by Lall et al. (2013). Briefly, 100 µl of HO-MeOH and tetracycline were serially diluted in BHI broth in a 96-well plate. Concentrations of plant extract ranged from 3.91 to 500 µg/ml, while tetracycline was tested from 0.39 to 50 µg/ml and was used as the positive control. Cultures of *C*. *acnes* grown for 72 h were then inoculated in BHI broth at a concentration of 1.5 × 10^8^ CFU/ml (OD_600_ = 0.132). To each test well 100 µl of bacterial suspension was added. The 96-well plates were then incubated in an Anaerocult^®^ jar with Anaerocult^®^ A for the generation of CO_2_. Control plates were included and consisted of *C. acnes* alone, an extract vehicle DMSO treatment (2.5% v/v), and a growth media control only. After 72 h, 20 µl of PrestoBlue^®^ reagent was added. The MIC (minimum inhibitory concentration) was then determined visually after 1 h of incubation. An MIC of less than 100 µg/ml was considered as potent anti-microbial activity.

#### Combinations of *Helichrysum odoratissimum* (L.) Sweet Methanol With Benzoyl Peroxide, Erythromycin, and Tetracycline

The interaction between the HO-MeOH (component A) extract and commercial agents (component B) were determined using the variable ratio method described by De Rapper et al. (2012). The commercial agents and HO-MeOH extract were prepared to a stock concentration of 2 mg/ml (10% DMSO v/v). In a 96-well micro-titer plate, 100 µl of BHI broth was added to all the wells. Each component (A and B) was then added in varying ratios (9A:1B; 8A:2B; 7A:3B; 6A:4B; 5A:5B; 4A:6B; 3A:7B; 2A:8B; and 1A:9B). The combined ratios were then serially diluted two-fold. Cultures of *C. acnes* grown for 72 h were then inoculated in BHI broth at a concentration of 1.5 × 10^8^ CFU/ml (0.132 at OD_600_). To each test well, 100 µl of bacterial suspension was added. The 96-well plates were then incubated in an Anaerocult^®^ jar with Anaerocult^®^ A for the generation of CO_2_. Control plates were included and consisted of *C. acnes* only (no treatment) and a solvent control (2.5% v/v DMSO). After 72 h, 20 µl of PrestoBlue^®^ reagent was added and following an hour incubation the MIC was determined. The MIC of HO-MeOH (component A) and antibiotic (component B) in combination, were then used to determine the sum of the fractional inhibitory concentrations (∑ FIC) using the following equation:

$$\sum \text{ FIC }=\text{ FIC A}+\text{ FIC B }=\;\frac{\text{MIC in combination}}{\text{MIC A alone}}+\;\frac{\text{MIC in combination}}{\text{MIC B alone}}$$

An FIC value ≤ 0.5 was considered a synergistic combination, an FIC of 0.5 ≤ 1 was considered as additive, an FIC of 1 < 4 was considered non-interactive, and finally an FIC > 4 was considered antagonistic.

#### Biofilm Prevention and Disruption

The bacterial adhesion assay for *C. acnes* (ATCC 6919) was previously performed by Coenye et al. (2007). Actively growing cultures of *C. acnes* were inoculated in sterile 96-well plates by adding 100 µl (0.132 at OD_600_) in BHI to each well. Following bacterial plating, 100 µl of plant extract and tetracycline were added to achieve concentrations of 0.24–31.25 and 0.02–3.13 µg/ml, respectively. Controls including 100% adhesion (untreated *C. acnes*) and 0% adhesion (BHI media only) were included. Plates were then incubated anaerobically for 72 h. Following incubation, the BHI media was removed, and plates were gently washed with 100 µl phosphate buffered saline (PBS) three times. Adhered cells were then fixed using 100 µl of 99% methanol for 15 min. The MeOH was aspirated, and plates were allowed to air dry for 20 min. Quantification of adhered cells was then performed by the addition of 100 µl of a 0.5% crystal violet for 20 min. The plates were then gently rinsed with distilled water to remove the excess crystal violet and allowed to air dry. The bound crystal violet was then dissolved using 160 µl of a 33% acetic acid solution. The optical density was then measured at 590 nm using a BIO-TEK Power-Wave XS multi-well reader (A.D.P., Weltevreden Park, South Africa). For the biofilm disruption, *C. acnes* were plated as described in the adhesion protocol. After the 72 h adhesion, BHI media was removed, and the plates were gently washed three times with PBS to remove planktonic cells. Following the wash step, 200 µl of fresh BHI was added, and plates were then incubated for an additional 72 h to allow for biofilm maturation. After incubation, the media was removed and replaced with 200 µl of the test sample in BHI broth. After 24 h of treatment, the biofilm was quantified using crystal violet.

#### Cytotoxicity Analysis

##### Human Immortalized Keratinocytes

HaCaT were cultured in Dulbecco’s modified Eagle’s medium (DMEM) supplemented with 10% fetal bovine serum (FBS), 100 µg/ml penicillin, 100 µg/ml streptomycin, and 250 µg/ml fungizone and incubated at 37°C and 5% CO_2_. Cells were grown to 80% confluency, in vented cell culture flasks (T-75) before subculture. Adhered cells were sub-cultured by cell detachment using trypsin-ethylenediaminetetraacetic acid (EDTA) followed by the addition of fresh media into new growing flasks. Cytotoxicity was determined using the XTT Cell Proliferation Kit II. In a 96-well plate, 100 µl of (1 × 10^6^ cells/ml) cell suspension was added, and cells were incubated for 24 h. The HO-MeOH extract was added at a concentration ranging from 3.125 to 400 µg/ml. Actinomycin D was used as the toxic inducer and was tested from 0.05 to 3.91 µg/ml. Treated cells were then incubated for 72 h. An untreated cell control and a 2% v/v DMSO vehicle control were also included. Following incubation, 50 µl of activated XTT was added to all the test and control wells and further incubated for 2 h. The optical density was read at 490 nm with a reference wavelength of 690 nm to remove background. Tests were performed in triplicate and the percentage viability was then calculated. The 50% inhibitory concentration (IC_50_) was then calculated using GraphPad Prism 4.0 software (Lall et al., 2013).

##### Murine Macrophage Cells (RAW264.7)

Murine macrophages (RAW264.7) cells were cultured in DMEM supplemented with 10% fetal bovine serum (FBS) and 1% penicillin-streptomycin and incubated at 37°C and 5% CO_2_. Cells were grown to 85% confluency in 100 mm cell culture dishes to prevent contact inhibition. Adhered cells were then sub-cultured by cell detachment using TrypLE followed by the addition of fresh media into new 100 mm cell culture dishes. Cytotoxicity was determined using the 3-(4,5-dimethylthiazol-2-yl)-2,5-diphenyltetrazolium bromide (MTT) reagent. In a 96-well plate, 100 µl of a 1 × 10^5^ cells/ml was added and incubated for 24 h. The HO-MeOH extract was tested at a concentration range from 3.91 to 125 µg/ml. A 5% DMSO (v/v) was added as the toxic inducer. An untreated cell control was also included in the assay. Following incubation, 20 µl of MTT (5 mg/ml in PBS) was added to the test and control wells and incubated for 4 h. This was followed by the addition of 200 µl of acidified isopropanol (0.1% HCl). Plates were covered in foil and placed on a rotating platform for 10 min. The optical density was then read at 560 nm with a reference wavelength of 670 nm to remove background. Tests were performed in triplicate, and the percentage viability was then calculated. The IC_50_ was then calculated using GraphPad Prism 4.0 software (Esposito et al., 2014).

#### Inhibition of Interleukin-1α

The effect of HO-MeOH on secreted IL-1α levels in human keratinocytes was investigated at non-lethal concentrations determined from the cell viability assay (3.125–400 µg/ml). Human keratinocytes were plated in a 24-well plate at a seeding density of 1 × 10^5^ cells per well and incubated for 24 h. The cells were stimulated with 100 µg/ml of heat-killed *C. acnes* and 100 ng/ml of LPS from *E. coli*, independently. The HO-MeOH was added together with the stimulating factor to determine inhibitory activity for 24 h. An untreated cell control, LPS only and *C. acnes* only controls were also included. Plates were incubated for 24 h in a humidified incubator with 5% CO_2_ and then centrifuged (980 rpm for 5 min) before the supernatant was collected, aliquoted, and stored at −80°C until use. The quantification of IL-1α was performed using an Elabscience ELISA kit (Biocom Africa, Pretoria, SA) according to the manufacturer protocol. The IL-1α concentration was calculated using a standard curve.

#### Inhibition of Lipase Activity

The inhibitory effect of HO-MeOH against lipase was determined described by Choi et al. (2003). Ellman’s reagent (DTNB) was dissolved in isobutanol to prepare a stock concentration of 40 mM. A stock concentration 10 mM of 2,3-dimercapto-1-propanol tributyrate (DMPTB) was then prepared in a solution of 6% Triton v/v, 50 mM Tris-Cl at a pH of 7.2. These were then stored at −20°C until the assay was performed. The lipase buffer was then prepared with 10 mM KCl and 10 mM Tris-Cl at pH 7.5 and was used to dissolve the lipase enzyme from *C. rugosa* (Sigma Aldrich, Johannesburg, South Africa). A stock solution of 0.5 M EDTA, 10% v/v Triton X-100, and 1 M Tris-Cl were also prepared and made up the reaction mixture. The reaction mixture consisted of the following volumes, 20 μl of 40 mM DTNB, 2 μl of 0.5 M EDTA, 5 μl of 10% v/v Triton X-100, and 50 μl of 1 M Tris-Cl, pH 7.5. The reaction mixture was added to a 15 ml centrifuge tube, and the volume adjusted to 900 μl with distilled water. For the enzyme inhibition assay, 160 μl of the reaction mixture was added to a 96-well plate with 10 μl of inhibitor (test sample in DMSO) and 20 μl of enzyme solution (10 U/ml). The plates were then incubated at 37°C for 5 min to allow for inhibitor-enzyme interaction. The reaction was initiated by the addition of 10 μl of a 4 mM DMPTB solution. For tests with no inhibitor, 10 μl of lipase buffer was added. The assay controls included enzyme and substrate (no inhibitor), DMSO (5% v/v) only, no DTNB, no DMPTB, and no enzyme (substrate only). The plated were then incubated for 30 min at 37°C in a BIO-TEK Power-Wave XS multi-well reader (A.D.P., Weltevreden Park, South Africa) while performing a kinetic read at 405 nm. The IC_50_ was then calculated using GraphPad Prism 4.0 software.

#### Anti-Inflammatory Targets Associated With Acne Progression

##### Pro-Inflammatory Cytokine Inhibition

The levels of pro-inflammatory cytokine (IL-1β, IL-6, IL-8, IL-10, IL-12p70, and TNF-α) production in HaCaT cells induced with heat-killed *C. acnes* was determined from cell culture supernatants using flow cytometry. The BD Cytometric Bead Array (CBA) Human Inflammatory Cytokine kit was used to detect cytokine production according to the manufacturer’s protocol (Cat. No. 551811) (BD Biosciences, San Jose, USA). HaCaT were seeded in 24-well plates at a density of 1 × 10^5^ cells/well in DMEM supplemented with 10% FBS, 100 µg/ml penicillin, 100 µg/ml streptomycin, and 250 µg/ml fungizone and incubated at 37°C and 5% CO_2_ for 24 h. After incubation, plating media was removed and replaced with 1 ml fresh culture media. Test samples were prepared in 1 ml of DMEM to yield final concentrations of 7.81 µg/ml (HO-MeOH), 0.78 µg/ml (tetracycline), *C. acnes* (100 µg/ml), and LPS (100 ng/ml) in test wells. The HO-MeOH was added at the MIC to *C. acnes*-stimulated cells to determine inhibition of cytokines produced by *C. acnes*. LPS-stimulation was used as a control for cytokine induction. Untreated cells were also included as a control. Following 24 h of incubation plates were centrifuges at 980 rpm for 5 min, and cell-free supernatants were collected and stored at −80°C until use. Cytokine analysis was performed using the Accuri C6 cytometer (BD, Biosciences, USA). Inhibition of cytokines was calculated as a percentage (%) reduction from cytokine levels in *C. acnes*-stimulated HaCaT cells.

##### Nitric Oxide Scavenging Activity and Intracellular Nitric Oxide Inhibition

The NO radical scavenging activity of HO-MeOH was determined using the methods described by Boora et al. (2014), adapted for a microtiter plate. In a 96-well plate, 90 μl of distilled water was added to the first row, and 50 μl of distilled water was added to the subsequent wells. A stock solution of HO-MeOH (10 mg/ml in methanol) was prepared. Serial dilutions of HO-MeOH were then prepared by adding 10 μl of the prepared stock solution to the first row and transferring 50 μl to subsequent wells. The test concentration of HO-MeOH ranged from 15.625 to 2,000 µg/ml. Ascorbic acid was used as the positive control and tested at the same concentrations. Methanol was used as the solvent control. The NO production was then initiated with the addition of 50 μl of 10 mM sodium nitroprusside to each well. To account for the effect of the color of the extract, an extract blank was prepared using distilled water. The plates were then incubated for 90 min at room temperature in the dark. For NO quantification, 100 μl of Griess reagent was added to all the wells and the OD_546_ was then determined immediately using the BioTek Power Wave XS (A.D.P. Veltevreden Park, South Africa). The IC_50_ was then calculated using GraphPad Prism 4.0 software.

The cellular NO scavenging activity of HO-MeOH using stimulated murine macrophages (RAW264.7) was performed according to the methods described by Esposito et al. (2014). Cell cultures were maintained as described above [*Murine Macrophage Cells (RAW264.7)*]. Cells were then seeded in a 96-well plate, at a density of (1×10^4^ cells per well) and allowed to attach for 24 h prior to treatment. The HO-MeOH was tested at non-lethal concentrations obtained from the MTT cell viability assay (3.91–31.25 µg/ml). The positive control dexamethasone was tested at 4 µg/ml (10 µM), and the negative control was DMSO (0.1% v/v). The cells were pre-treated with test samples for 2 h before the induction of NO with 1 µg/ml of LPS (*E. coli* 0127: B8). Plates were then incubated overnight at 37°C in a humidified incubator with 5% CO_2_. The amount of NO produced in treated cells was detected using 50 µl of cell supernatant using the manufacturer’s protocol described in the Promega Griess Reagent System (Promega Corporation, WI, USA). The optical density was then read at 530 nm using a BioTek Synergy H1 microplate reader. The IC_50_ was then calculated using GraphPad Prism 4.0 software.

##### Cyclooxygenase-II Inhibition

Inhibition of human recombinant COX-II activity was investigated using the methods described by Reininger and Bauer (2006). Briefly, 5 µl of COX-II (0.5 U/well) was added to 180 µl of 100 mM Tris buffer (pH 8.0) containing cofactors as follows: 5 µM porcine hematin, 18 mM L-epinephrine, and 50 µM Na_2_EDTA in a 96-well plate. Stock concentrations of HO-MeOH and Ibuprofen were prepared in DMSO, and 10 µl of inhibitor was added to obtain final concentrations of 2.5–160 µg/ml for HO-MeOH and 0.4–10 µM (0.01–0.15 µg/ml) for ibuprofen, the positive control. After 5 min incubation with the test inhibitors, 5 µl of 10 µM arachidonic acid substrate was added to initiate the reaction. The test plate was incubated at 37°C for 20 min. Following incubation, 10 µl of 10% formic acid was added as a stop solution. The production of PGE_2_ was quantified using the PGE_2_ ELISA kit according to the manufacturer’s protocol (Cat. No. ADI-900-001) (Enzo Life Sciences, New York, USA). The quantification of PGE_2_ in test inhibitors and controls was determined from the PGE_2_ standard curve. The IC_50_ values for test inhibitors were calculated using GraphPad Prism 4.0 software.

##### Inhibition of Inflammatory Gene Expression

Murine macrophages (RAW264.7) were maintained as in the section *Murine Macrophage Cells (RAW264.7)*.** Cells were seeded in a 24-well plate at a cell density of 5 × 10^5^ cells/ml and incubated for 24 h in a humidified incubator with 5% CO_2_. Cells were then treated with non-lethal concentrations of HO-MeOH (3.91–31.25 µg/ml) and dexamethasone was used as the positive control tested at 4 µg/ml (10 µM). Treatments with the extract and positive control were performed for 2 h before the addition of 1 µg/ml of LPS. Controls with untreated cells, 0.1% v/v DMSO and LPS only were also included. Plates were then incubated overnight. The total RNA was then isolated using the TRIzol reagent. Briefly, 330 µl of TRIzol was added to each replicate and combined in a sterile Eppendorf tube. Samples were then frozen at −20°C overnight. Following incubation, samples were thawed before the addition of 200 µl chloroform and vortexing for 30 s. Eppendorf tubes were then centrifuged (12,000 g) for 15 min. Samples were then carefully removed and the clear upper layer (~400 µl) was pipetted and transferred to a clean, sterile Eppendorf tube, without disturbing the pink TRIzol layer. To the supernatant layer, 500 µl of ice-cold isopropanol was added, vortexing briefly and storing on ice for 10 min. Samples were then centrifuged and supernatants discarded. Pellets were washed with 1 ml of 70% ethanol (using absolute ethanol and molecular grade water) followed by brief vortexing and storage on ice for 10 min before centrifuging (7,500 g) for 5 min. The supernatant was decanted without disturbing the pelleted RNA pellet. The Eppendorf tubes were air-dried for 15–20 min to allow residual ethanol to evaporate. Samples were stored in 40 µl of molecular grade water and stored at −80°C until use. RNA was quantified using the BioTek Synergy H1/Take 3 spectrophotometer to determine the OD_260_/OD_280_ ratio. The cDNA was synthesized using 2 µg for each test sample and the controls using the cDNA Reverse Transcriptase Kit (Life Technologies, California, USA) on the ABI Applied Biosystems GeneAMP 9700 thermal cycler (Life Technologies, California, USA). Synthesized cDNA was amplified in duplicate using real-time quantitative PCR using SYBR green PCR Master Mix (Life Technologies, California, USA). The effects of genomic DNA contamination were overcome by selective use of intron-overlapping primers selected using Primer Express version 2.0 software (ABI Applied Biosystems, California, USA). The quantitative PCR amplifications were performed using the ABI Applied Biosystems 7500 Fast Real-Time PCR System (Life Technologies, California, USA) with 1 cycle at 50°C for 2 min and 1 cycle at 95°C for 10 min, subsequently followed by 40 cycles of 15 s at 95°C and 1 min at 60°C. Dissociation curves were completed with a single cycle of 1 min at 95°C, 30 s at 55°C, and finally 30 s at 95°C. The mRNA expression was determined using ∆∆CT analysis with data normalized against the β-actin housekeeping gene using the 7500 Fast System SDS software v1.3.0. Inhibition if gene expression was compared with LPS-stimulated gene expression, where values < 1 indicated inhibition of gene expression and values > 1 indicated the overexpression of the gene of interest in excess of that of LPS stimulation. Melting curve profiles for the specific transcripts were also obtained. The forward primers sequences for each gene were as follows: β-actin (5′-AAC CGT GAA AAG ATG ACC CAG AT-3′), COX-II (5′-TGG TGC CTG GTC TGA TGA TG-3′), and inducible nitric oxide synthase (iNOS) (5′-CCC TCC TGA TCT TGT GTT GGA-3′). The reverse primers for each gene were as follows: β-actin (5′-CAC AGC CTG GAT GGC TAC GT-3′), COX-II (5′-GTG GTA ACC GCT CAG GTG TTG-3′), and iNOS (5′-TCA ACC CGA GCT CCT GGA A-3′) (Integrated DNA Technologies, Illinois, USA).

##### Inhibition of Hyaluronidase Activity

The turbidometric assay described by Hofinger et al. (2007) was used to determine the hyaluronidase inhibitory activity. A citrate-phosphate buffer (McIlvaine’s buffer) was prepared by mixing a solution of 0.2 M Na_2_HPO_4_ with 0.1 M NaCl and 0.1 M citric acid with 0.1 M NaCl to pH 5.0. The hyaluronic acid substrate stock solution was prepared to 2 mg/ml (in distilled water). A stock solution of 0.2 mg/ml (in distilled water) bovine serum albumin (BSA) was also prepared. The hyaluronidase from *Streptococcus pyogenes* was optimized to 1 U/ml. In a 96-well plate, 136 μl of citrate-phosphate buffer was added to the first row. To all the subsequent wells 70 μl of buffer was added. The HO-MeOH extract was diluted in DMSO, and 4 μl was added to the first row in triplicate, followed by a two-fold serial dilution to obtain final concentrations ranging from 1.95 to 250 µg/ml. Ten microliters (10 μl) of BSA and 10 μl of hyaluronidase enzyme were then added. The negative control was DMSO without sample. The plates were then incubated for 15 min at 37°C. The reaction was then initiated by the addition of 10 μl of hyaluronic acid and further incubation for 30 min at 37°C. The reaction was then stopped with the addition of 200 μl of 2.5% (w/v) cetyltrimethylammonium bromide (CTAB) in 0.5 M NaOH and incubated at room temperature for 20 min. The turbidity was then measured by obtaining the optical density at 355 nm using the BioTek PowerWave XS (A.D.P. Veltevreden Park, South Africa). Additional controls included an extract color control, a substrate only control (100% enzyme inhibition), an enzyme and substrate only control (0% inhibition), and a no substrate control. The IC_50_ was then calculated using GraphPad Prism 4.0 software.

### Statistical Analyses

Results for each experiment were determined using data obtained in triplicate with three independent assay repeats. One-way analysis of variance (ANOVA) was used to determine the differences in means and the Dunnett’s multiple comparison post-test was used to determine significant differences between the means of treated and untreated groups as identified in each bioassay. For enzyme assays a four parameter logistic equation with a 95% confidence interval was performed to determine the IC_50_.

## Results and Discussion

### Gas Chromatography–Mass Spectrometry and Liquid Chromatography–Mass Spectrometry Analysis

The major constituents in the extract were oleic acid amide (11.33%), linoleic acid (9.16%), palmitic acid (8.52%), caryophyllene (8.13%), oxalic acid allyl nonyl ester (8.13%), 5,5-diethoxy-2-pentanone (4.74%), stigmasterol (4.36%), *α*-sitosterol (4.28%), *α*-humulene (3.94%), *α*-curcumene (3.74%), and valencen (3.23%) (**Table 1**) (**Figure S1**). Lower levels of linoleic acid have been detected in acne patients. This fatty acid is of particular interest as it cannot be synthesized in human body and is generally obtained through diet. In sebaceous gland cells, low levels of linoleic acid influence linoleate concentrations in sebum, which translate to a fatty acid deficiency in the cells of the follicular epithelium leading to hyperkeratinization (Downing et al., 1986; Makrantonaki et al., 2011). This could contribute to the treatment of comedonal acne. The presence of stigmasterol and *α*-sitosterol may contribute to HO-MeOH biological activity as previously isolated plant sterols isolated from green tea have shown therapeutic effects for acne vulgaris (Saric et al., 2016).

**Table 1:** Compounds present in the methanolic extract of *Helichrysum odoratissimum* identified through gas chromatography–mass spectrometry.

Retention time (min)	Abundance (%)	Formula	Molecular weight (g/mol)	Compound name
6.24	1.9744	C_9_H_20_	128	Pentane, 2,2,4,4-tetramethyl-
7.07	2.1806	C_7_H_5_NS	135	Benzothiazole
7.23667	2.0781	C_8_H_14_O_2_	142	2,7-Octanedione
5.32667	0.84052	C_8_H_18_O_2_	146	Propane, 1,1-diethoxy-2-methyl-
6.77167	1.9106	C_11_H_24_	156	Undecane
6.13	4.742	C_9_H_18_O_3_	174	2-Pentanone, 5,5-diethoxy-
10.3967	3.7395	C_15_H_22_	202	α-Curcumene
11.5583	3.2313	C_15_H_22_	202	Valencen
8.99333	2.0484	C_15_H_24_	204	à-Cubebene
9.94667	3.9443	C_15_H_24_	204	Humulene
9.505	8.1263	C_15_H_24_	204	Caryophyllene
12.0883	1.7008	C_15_H_24_O	220	11,11-Dimethyl-4,8-dimethylenebicyclo[7.2.0]undecan-3-ol
22.3183	1.8196	C_15_H_26_O	222	trans-Farnesol
15.2583	0.6965	C_14_H_28_O_2_	228	Tridecanoic acid, methyl ester
15.685	8.5215	C_16_H_32_O_2_	256	Palmitic acid
9.51667	8.1263	C_14_H_24_O_4_	256	Oxalic acid, allyl nonyl ester
15.3767	1.301	C_20_H_30_	270	Gerany-P-cymene
17.1767	9.1583	C_18_H_32_O_2_	280	Linoleic acid
22.0833	11.329	C_18_H_35_NO	281	Oleic acid amide
16.91	2.2018	C_20_H_40_O	296	Phytol
17.6117	2.0086	C_20_H_40_O_2_	312	Butyl palmitate
19.2683	2.9919	C_22_H_42_O_4_	370	Hexanedioic acid, bis(2-ethylhexyl) ester
19.385	1.8204	C_27_H_56_	380	Heptacosane
24.7767	4.3613	C_29_H_48_O	412	Stigmasterol
23.365	0.45973	C_26_H_38_O_4_	414	Lupulon
23.4467	2.2776	C_26_H_38_O_4_	414	Lupulon
22.9983	2.1277	C_27_H_55_Cl	414	Heptacosane, 1-chloro-
25.135	4.282	C_29_H_50_O	414	á-Sitosterol

The HO-MeOH shared common compounds with other *Helichrysum* species, including *Helichrysum aureonitens* (*α*-humulene and *para*-cymene). Hydrodistilled *Helichrysum leucocephalum* and *Helichrysum artemisiodes* led to the identification of *β*-caryophyllene and *α*-humulene as major components of the oils of both species. These species also contained other constituents like hexadecanoic acid and E, E-farnesene present in HO-MeOH (Yani et al., 2005; Javidnia et al., 2009). A study by Bougatsos et al. (2003) reported that *Helichrysum kraussii* and *Helichrysum rugulosom* both contained *β*-caryophyllene as a major constituent. The *H. kraussii* oil also contained 9.8% *α*-humulene, which was present in HO-MeOH. This study also reported the antibacterial activity of the individual component *β*-caryophyllene against *Staphylococcus aureus* and *Staphylococcus epidermidis* and showed that the crude essential oil exhibited better activity. This could explain the potent antimicrobial activity of HO-MeOH. Kuiate et al. (1999) reported the presence of *α*-curcumene and *β*-caryophyllene in *H. odoratissimum* L. Less. This study also reported the presence of *α*-humulene in smaller quantities. Gundidza and Zwaving (1993) reported similar results in a Zimbabwean *H. odoratissimum* species with *α*-humulene (13%) and *β*-caryophyllene (9.6%) as a major components. Valencene (1.9%), *para*-cymene (0.3%), and *α*-cubebene (0.1%) were also present in the oil showing good correlation with the HO-MeOH components. Lawal et al. (2015) who tested a South African *H. odoratissimum* L. Sweet species, found that *α*-humulene (5.6%) and *β*-caryophyllene (4.7%) were the major sesquiterpene compounds present.

The LC-MS data of the negative ionization mode, identified a caffeoylquinic acid derivative as a possible constituent in the crude methanolic extract (**Table 2**, **Figure S2** and **Figure S3**). The identified mass fragment m/z 515.1223 making up the peak at 3.71 min (**Figure S4**—**Supplementary Data**) was of particular interest since the possible compound hits included some caffeoylquinic acid derivatives which have been previously identified in other *Helichrysum* species, including *Helichrysum italicum*, *Helichrysum populifolium*, and Helichrysum *stoechas* (Barroso et al., 2014; Heyman et al., 2015; Maksimovic et al., 2017; Pereira et al., 2017). The mass fragments m/z 353.1017 and 191.0762 corresponded to previous data on Helichrysum *obconicum* reported by Gouveia and Castilho (2011) corresponding to some caffeoyl-quinic acid derivatives. The 4,5-O-dicaffeoylquinic acid derivative was determined as the most probable compound match after comparison of the MS fragmentation pattern given for this derivative on the METLIN database (**Figure S5**).

**Table 2:** Identified compound match present in the methanolic extract of *Helichrysum odoratissimum* using negative ionization mode liquid chromatography–mass spectrometry data.

Peak	Retention time	Molecular mass (g/mol)	Elemental composition from MassLynx	Corrected mass fragments (m/z)	Potential compound match
1.	3.71	516.40	C_25_H_24_O_12_	515.1223 353.1017 191.0762	4,5-Di-O-caffeoylquinic acid 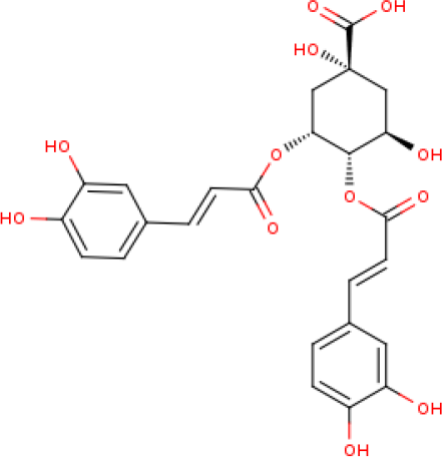

### Antimicrobial Activity and Interaction With Benzoyl Peroxide, Erythromycin, and Tetracycline

The antibacterial activity of the methanolic leaf and stem extract of *H. odoratissimum* (HO-MeOH) exhibited an MIC of 7.81 µg/ml against *C. acnes*, while the positive control tetracycline, exhibited an MIC of 0.78 µg/ml. The minimum bactericidal concentration (MBC) activity was also determined with the HO-MeOH exhibiting a bactericidal effect at 250 µg/ml, compared with tetracycline at 1.56 µg/ml. The effect of the combination of HO-MeOH and the macrolide antibiotic, erythromycin, was also antagonistic to a much larger degree. The general trend observed for the combination of HO-MeOH and benzoyl peroxide (BPO) was that lower concentrations of HO-MeOH (sub-MIC) meant that less BPO was needed to produce an antimicrobial effect. The MIC of BPO alone was 31.25 µg/ml, but with the addition of the HO-MeOH extract in three combinations, antimicrobial activity of BPO was observed at concentrations as low as 5.47, 6.25, and 7.03 µg/ml (**Table 3**).

**Table 3:** Biological activity of *Helichrysum odoratissimum* methanolic extract against important targets of *Cutibacterium acnes* associated with acne progression.

Sample	MIC (µg/ml)	MBC (µg/ml)	Bacterial adhesion IC_50_ (µg/ml)	Biofilm disruption IC_50_ (µg/ml)	Nitric oxide scavenging IC_50_ (µg/ml)	COX-II IC_50_ (µg/ml)	Lipase IC_50_ (µg/ml)	Hyaluronidase IC_50_ (µg/ml)
HO-MeOH	7.81	250	<0.24	7.65 ± 0.60	214.90 ± 13.27	22.87 ± 6.48	157.50 ± 6.85	145.45 ± 6.22
Combination 1: 3:7 dilution 7	2.34 HO 5.47 BPO	*0.48	–	–	–	–	–	–
Combination 2: 2:8 dilution 7	1.56 HO 6.25 BPO	*0.40	–	–	–	–	–	–
Combination 3: 1:9 dilution 7	0.78 HO 7.03 BPO	*0.33	–	–	–	–	–	–
L-ascorbic acid	–	–	–	–	35.24 ± 7.85	–	–	–
Benzoyl peroxide	31.25	–	–	–	–	–	–	–
Erythromycin	0.06	–	–	–	–	–	–	–
Ibuprofen	–	–	–	–	–	0.15 ± 0.002	–	–
Tetracycline	0.78	1.56	0.32 ± 0.11	>3.125	–	–	>50	>250

*ΣFIC (sum fractional inhibitory concentration) for synergistic combinations of HO-MeOH and BPO showing synergistic antibacterial activity (ΣFIC < 0.5).

The antimicrobial activity of *H. odoratissimum* has, however, been tested previously against other microorganisms that have been linked to acne progression. The antibacterial activity of 3-*O-*methylquercetin isolated from the methanolic flower extract of *H. odoratissimum* showed selectivity against the Gram-positive bacteria *Bacillus subtilis* and *S. aureus* with an MIC of 50 and 6.25 µg/ml, respectively (Van Puyvelde et al., 1989). Although the flowers were not included in this study, a review by Lourens et al. (2008) indicated the presence of other flavonoid derivatives including chalcones and flavonols found in the aerial parts and roots of *H. odoratissimum.* The presence of pyrone, phloroglucinols, and diterpenes were mentioned and could be responsible for the observed antimicrobial activity against Gram-positive *C. acnes*. The antimicrobial activity of the acetone extract of *H. odoratissimum* also showed antimicrobial activity against *S. aureus,* with an MIC of 15.63 µg/ml (Lourens et al., 2004). The antimicrobial activity of *H. odoratissimum* against *C. acnes*, was more potent when compared to other *Helichrysum* species. *Helichrysum pallidum*, *H. aureonitens*, and *H. splendidum* showed poor inhibition of the bacterium with MICs at ≥ 500 µg/ml, while *H. kraussii* exhibited an MIC of 61.25 µg/ml (De Canha et al., 2018; Lall et al., 2019).

The acetone shoot extract of *H. odoratissimum* which excluded the flowers was prepared by Mathekga and Meyer (1998) and reported antibacterial activity against *S. aureus* and *B. subtilis* at 10 µg/ml. The choloform:methanol (1:1) leaf and stem extract of *H. odoratissimum* exhibited no activity against *S. epidermidis*, another microorganism which has been implicated in the pathogenesis of acne with an MIC of 4 mg/ml but was active against *S. aureus* with an MIC of 20 µg/ml (Lourens et al., 2011; Kumar et al., 2016). Previous reports on the antimicrobial activity of *H. odoratissimum* suggested that this plant contains compounds that are more selective toward Gram-positive bacteria, and showed better activity against *C. acnes* when compared to other acne related Gram-positive microorganisms used in previous studies. These results provide scientific evidence for the application of the extract to pimples, as it potentially reduces *C. acnes* load within the acne lesions.

Antibiotic resistance is often linked with extensive use of antibiotics as a treatment option. The topical use and oral administration of antibiotics are common practice in acne treatment. Reports suggest that more than 50% of clinical isolates of *C. acnes* are resistant to topical macrolide antibiotics such as erythromycin. The use of broad-spectrum antibiotics also disturbs the delicate balance of the skin microbiome and can promote the growth of opportunistic bacteria. The use of topical compounds such as benzoyl peroxide (BPO), has therefore been prescribed as a solution for using antibiotic monotherapies or concurrent topical and oral antibiotic therapies in isolation. This compound is often used when long-term antibiotic use is prescribed for the treatment of acne patients. While macrolide antibiotics are the most common topical antibiotics, cyclines are the most common orally prescribed antibiotics. The burden of antibiotic resistance is escalated by the long treatment regimens often lasting between 3 and 6 months (Walsh et al., 2016). The combination of the HO-MeOH extract with erythromycin, tetracycline, and BPO was investigated to determine what type of interaction occurs and whether the interaction could potentiate antimicrobial activity against *C. acnes*. The MICs for erythromycin and BPO were determined independently and were shown to inhibit *C. acnes* growth at 0.061 and 31.25 µg/ml, respectively. The HO-MeOH extract showed an overall antagonistic effect when combined with the cycline antibiotic, tetracycline. The general trend of the results suggested that a decrease in HO-MeOH will always require more antibiotic to show the same antimicrobial activity (**Table 2**).

The tested *C. acnes* strain ATCC 6919 was more susceptible to erythromycin than to tetracycline and BPO. Macrolides such as erythromycin can reversibly bind to the 50S ribosomal subunit of bacteria and inhibit protein translocation, and tetracycline binds to the 30S ribosomal subunit preventing the synthesis of proteins essential for bacterial cell function (Chukwudi, 2016; Fox et al., 2016). The compounds present in the HO-MeOH extract could potentially interfere with the binding of tetracycline and erythromycin to the ribosomal subunits, decreasing their antimicrobial activity. Studies investigating the *in vitro* effects of combinations of antibiotics and plant extracts on *C. acnes* specifically, are not common. However, there are numerous scientific reports of combinations of plant essential oils and antibiotics on other acne-associated bacteria.

Oliveira et al. (2006) reported antagonistic effects for combinations of the essential oils of *Plectranthus amboinicus* and *Eucalyptus citriodora* with gentamicin against *S. epidermidis*. Combinations of *E. citriodora* oils with gentamicin, chloramphenicol, and ampicillin were all antagonistic against *S. aureus*. Several antagonistic effects were also observed for combinations of the oils of *Lippia sidoides* with gentamicin, *P. amboinicus* with chloramphenicol and gentamicin and *Conyza bonariensis* with chloramphenicol and gentamicin with regards to antimicrobial activity against *S. aureus*. Although it is an aminoglycoside antibiotic, gentamicin, much like tetracycline, affects protein synthesis by binding to the 16S A-site, which forms part of the 30S ribosomal subunit (Kotra et al., 2000). A study by Van Vuuren et al. (2009) also showed the antagonistic effects of commercial tea tree essential oil when combined with ciprofloxacin against *S. aureus*, using the ratio method. *H. odoratissimum* is a highly aromatic species with several volatile constituents such as oxygenated monoterpenes (Asekun et al., 2007) which could potentially explain the antagonistic activity. The emergence of resistant strains of bacteria has encouraged the investigation of combinations of current antibiotic compounds with plant extracts and plant compounds. However, many of these reports focus on the publication of data where positive combinations are observed while ignoring the relevance of potentially antagonistic combinations (Sibanda and Okoh, 2007).

Benzoyl peroxide itself does not show potent antimicrobial activity, according to previously reported studies on 44 clinical isolates of *C. acnes* the MIC of BPO ranged between 128 and 256 µg/ml for all the isolates (Okamoto et al., 2016a; Okamoto et al., 2016b). In this study, the MIC was reported as 31.25 µg/ml against the type strain of *C. acnes* (ATCC 6919), which is particularly more susceptible to antimicrobial compounds. The potent antimicrobial activity of erythromycin can also be used to explain the susceptibility of the tested strain because clinical isolates of *C. acnes* commonly have higher resistance toward both erythromycin and clindamycin (Dessinioti and Katsambas, 2017). It is therefore expected that BPO would show a lower MIC against the type strain. This study showed that three concentrations of HO-MeOH resulted in better synergistic activity by keeping the BPO concentrations well below its MIC. The antibacterial activity of BPO is based on the breakdown of this compound into benzoic acid and hydrogen peroxide oxidative free radicals. These are highly reactive compounds that can affect *C. acnes* growth by damaging cellular constituents (Kosmadaki and Katsambas, 2017). Plants from the *Helichrysum* genus are known to possess many anti-oxidant compounds which can neutralize reactive oxygen species. The constituents found in the HO-MeOH extract could, therefore, be interacting with oxidative free radicals produced by the breakdown of the peroxide bond in BPO, which is known to be very unstable. This could potentially explain why at lowered concentrations of HO-MeOH show better synergistic potential (Albayrak et al., 2010). In this study the mechanism of antimicrobial activity was not tested. However, it is possible that the HO-MeOH extract targets the synthesis of enzymes responsible for protection against oxidative stress, resulting in increased susceptibility to BPO generated radicals (Nusbaum et al., 2012). It is important to note that combining components to achieve a desired effect; in this case, antimicrobial activity, is highly dependent on the selection of specific concentrations of each component.

### Biofilm Prevention and Disruption

**Figure 1:**
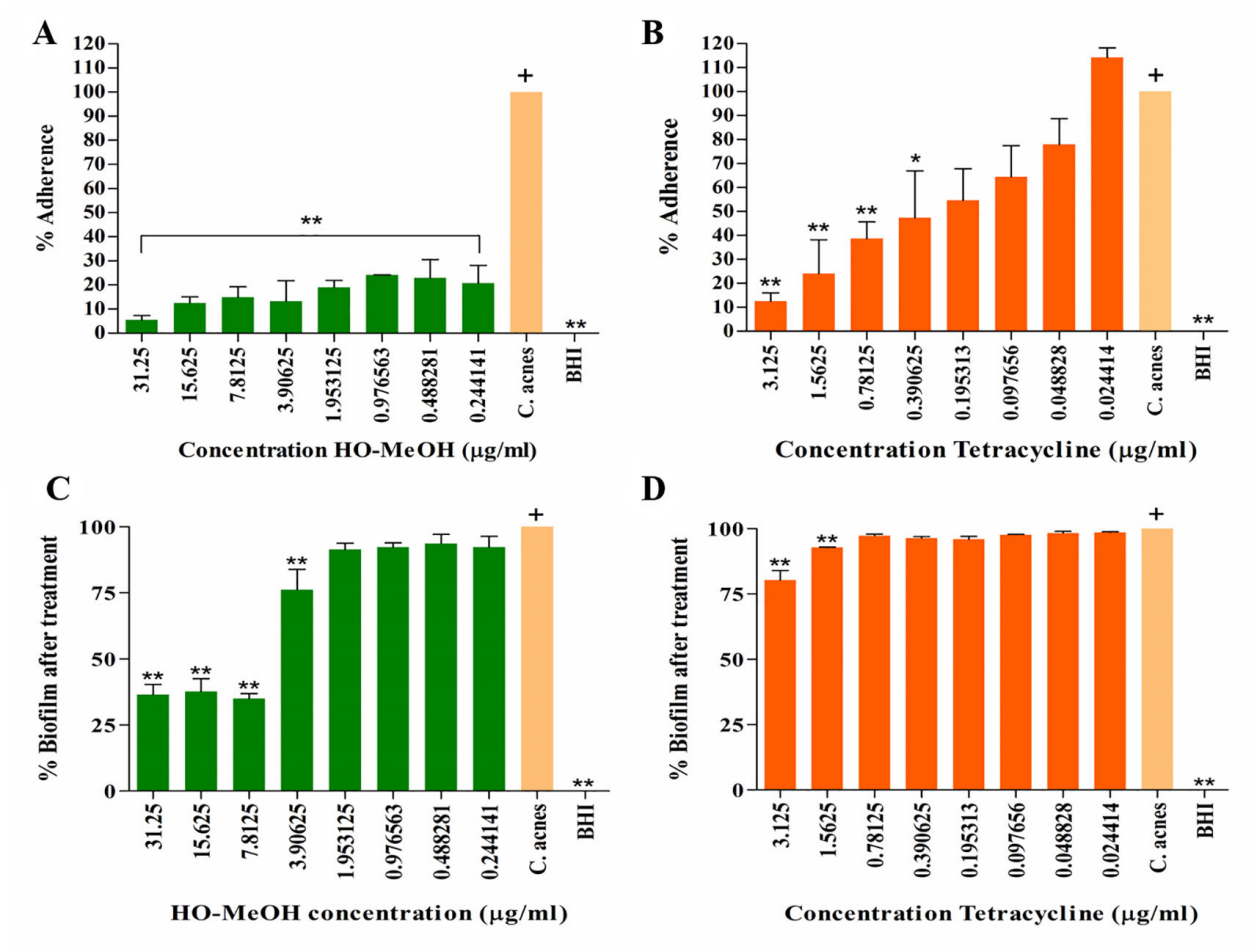
The anti-adherence activity of the *Helichrysum odoratissimum* methanolic extract **(A)** and tetracycline **(B)** and the biofilm disruption activity of the *H. odoratissimum* methanolic extract **(C)** and tetracycline **(D)**. *p < 0.05; **p < 0.01. The “+” indicates the control column to which all the data sets were compared using the Dunnet’s multiple comparison test.

A study by Coenye et al. (2007) reported the ability of the ATCC 6919 strain of *C. acnes* to attach to polystyrene prior to the development of a mature biofilm *in vitro*. The HO-MeOH extract exhibited high anti-adherence activity even at concentrations well below the MIC. Inhibition of attachment observed at 15.63 and 31.25 µg/ml is most likely due to the antimicrobial activity affecting *C. acnes* growth. All the concentrations of HO-MeOH showed statistically significant differences when compared with the *C. acnes* untreated control, which was defined as the 100% adherence (**Figure 1A**). The BHI only control retained no crystal violet and indicated that no contaminants were present in the growth media. Bacterial adherence is often due to charge, hydrophobicity, or production of extracellular polysaccharides of cells (Zeraik and Nitschke, 2012). A study by Nostro et al. (2004), which tested the effect of *Helichrysum italicum* on the adherence of *Streptococcus mutans*, observed that the extract was able to reduce adherence by causing changes in the hydrophobicity index to a hydrocarbon surface. Styrene, the component making up the polystyrene plates, is itself a hydrocarbon and therefore, suggests that the attachment of *C. acnes* to this surface is due to hydrophobic bonding (Rosenberg, 2017). The formation of biofilm is largely dependent on the ability of the microorganism to adhere to the substrate or surface. This adhesive ability is, therefore, one of the targets available for the prevention of biofilm formation and is of particular importance, because mature biofilm structures play a crucial part in disease development.

Studies on the polar constituents of some *Helichrysum* species through hydro-methanolic, hydro-alcoholic, or methanol have identified caffeoylquinic and flavonoid derivatives as major constituents (Mari et al., 2014; Silva et al., 2017). The quinic acid derivative chlorogenic acid isolated from the methanol extract of *H. italicum* exhibited anti-adhesive activity against Pseudomonas *aeruginosa* (D’Abrosca et al., 2013). The results observed in this study suggested that HO-MeOH contained an active constituent or a number thereof, which could potentially disrupt hydrophobic interactions between the polystyrene surface and the *C. acnes* cells. The drug control tetracycline was also tested for its ability to inhibit the attachment of *C. acnes*. Tetracycline was not as effective as the HO-MeOH extract with regards to the inhibition of bacterial cell adhesion. Tetracycline exhibited an IC_50_ of 0.32 ± 0.11 µg/ml (**Figure S8A**), which indicates anti-adhesion activity was not due to bacterial cell death as this was below the MIC of 1.56 µg/ml (**Figure 1B**). The results suggested that tetracycline is used solely for its ability to inhibit excessive proliferation of the bacterial cells, targeting protein synthesis. In a clinical setting, *C. acnes* thrives within a biofilm and often results in reduced activity of antibiotic therapies. The addition of sub-MIC concentrations of tetracycline does not inhibit the attachment of *C. acnes* cells as well as the HO-MeOH. Tetracycline tested at a sub-inhibitory concentration of 0.02–0.2 µg/ml was not able to inhibit biofilm formation after 72 h of treatment, suggesting that bacterial adhesion and maturation remains unaffected by this antibiotic (Sivasankar et al., 2016).

Gram-negative and Gram-positive bacterial populations are known to cooperate, communicate, and grow symbiotically to form complex social communities in order to enhance invasion and persistence through a process known as quorum sensing. The biofilm structure forms an integral part of this process and is, therefore, an essential target to consider when developing acne therapies, considering the ineffectiveness of systematic antibiotics in this regard (Savoia, 2012). There was disruption of mature *C. acnes* biofilm at 31.25, 15.625, and 7.81 µg/ml when compared to the untreated *C. acnes* control. The IC_50_ for the biofilm disruption was calculated to be 7.65 ± 0.60 µg/ml (**Figure 8B**). The extract also showed disruption of the *C. acnes* biofilm at a sub-inhibitory concentration of 3.91 µg/ml, suggesting that the extract does not act only by killing cells within the biofilm but may have an additional role (Coenye et al., 2012) (**Figure 1C**). When compared with other extract tested against *C. acnes* biofilm disruption, *H. odoratissimum* showed prominent activity. A study by Chassagne et al. (2019) reported the biofilm eradication concentration (MBEC_50_) of *Gingko biloba* seed nut extract against *C. acnes* at 256 µg/ml and an MIC of 64 µg/ml.

The crystal violet staining method effectively allows for the quantification of biofilm biomass but is limited; however, by the inability to distinguish between live and dead cells (Peeters et al., 2008). Although the specific mechanism of biofilm disruption was not tested in this study, the results suggest that the biofilm disruption effect of HO-MeOH is most likely due to the bactericidal activity against cells within the biofilm. The majority of compounds possessing anti-biofilm activity are isolated from natural sources (Roy et al., 2018). A study by Cui et al. (2016) observed not only the inhibitory effect of the essential oil of *H. italicum* against biofilm formation but also its ability to eradicate biofilm structure of *S. aureus*. The essential oil showed better activity in both aspects when compared with the effect of major essential oil component, neryl acetate, alone. This suggested that the essential oil components act synergistically against biofilm formation and biofilm disruption. The HO-MeOH is contains a number of volatile compounds identified through GC-MS, which could act synergistically resulting in the disruption of *C. acnes* biofilm. An amino-phloroglucinol, helichrytalicine B, isolated from *H. italicum* showed potent activity against the formation of *S. epidermidis* biofilms. Many *Helichrysum* species are abundant in α-pyrone phloroglucinol derivatives, including *H. odoratissimum* which could be responsible for its biofilm eradication activity. Due to the ability of biofilm-forming bacteria to alternate between both single cell and biofilm states, it is important for agents to possess both strong anti-biofilm activity as well as antibacterial activity. This makes HO-MeOH a promising candidate as an anti-biofilm agent (Lourens et al., 2008; D’Abrosca et al., 2016). Tetracycline was less effective at disrupting established *C. acnes* biofilm. There was only significant biofilm disruption compared to the untreated *C. acnes* control at 1.56 and 3.125 µg/ml (**Figure 1D**). Biofilm structures are known to result in increased resistance to antibiotic activity; this could explain the ineffectiveness of tetracycline to reduce biofilm mass after treatment of the mature biofilm. Other tetracycline antibiotics (doxycycline and oxytetracycline), except minocycline, were not able to reduce biofilm biomass or exert bactericidal activity against *C. acnes* cells within the biofilm (Coenye et al, 2007). Biofilm formation varies between different bacterial phylotypes, ATCC 6919 has been classified as type IA_1_. This phylotype can produce higher amounts of biofilm biomass, possibly contributing to reduced efficacy of tetracycline (Kuehnast et al., 2018).

### Cytotoxicity on Human Keratinocytes and Murine Macrophages

**Figure 2:**
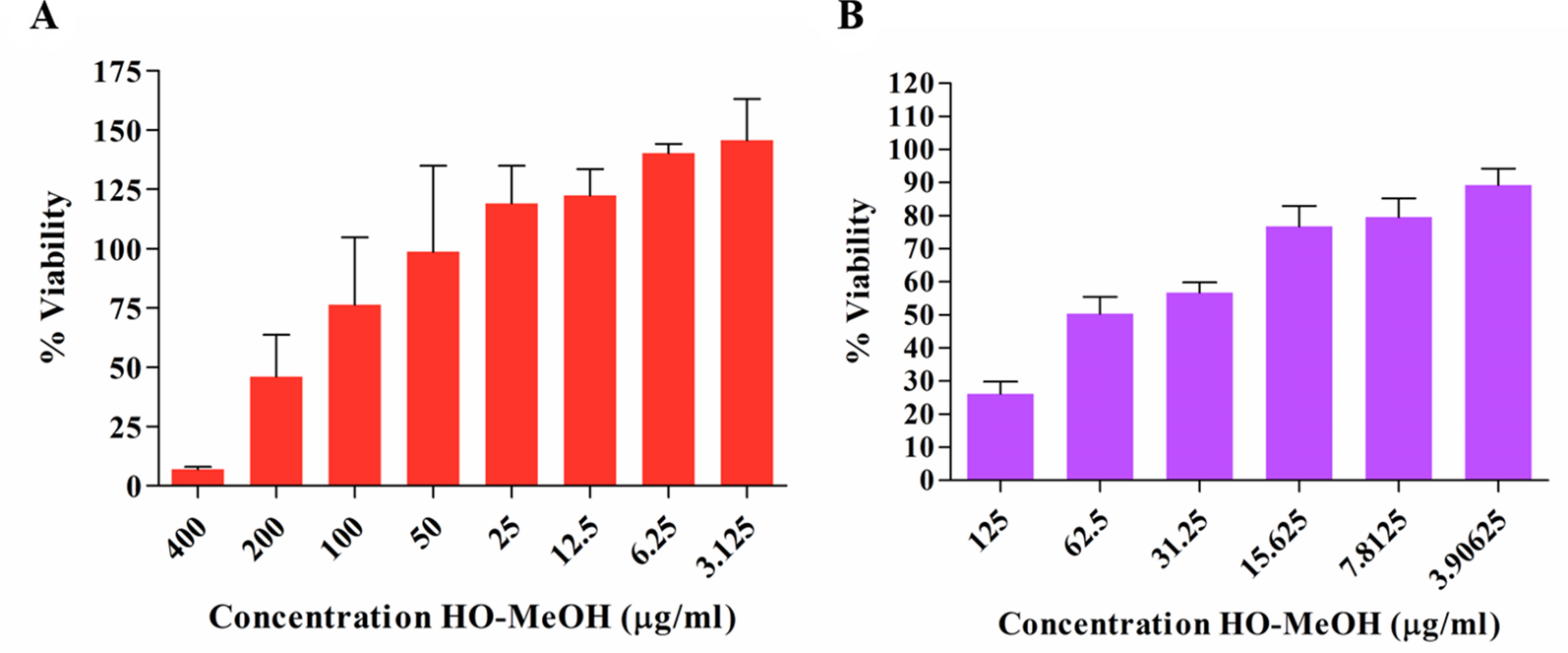
Antiproliferative effects of the *Helichrysum odoratissimum* methanolic extract on human keratinocyte cells (HaCaT) **(A)** and murine macrophage cells (RAW264.7) **(B)**.

The anti-proliferative effect of HO-MeOH was determined on human keratinocyte cells in order to determine non-lethal concentrations of the extract that could be investigated in subsequent hyper-keratinization and anti-inflammatory cell-based assays. Cell viability was normalized against the cell viability observed in the DMSO negative control. The IC_50_ of HO-MeOH on HaCaT viability determined to be 167.00 ± 27.95 µg/ml (**Figures 2A** and **S9A**). This is well above the MIC, suggesting that topical application of the extract at the MIC concentration should not affect HaCaT cell viability. Several *Helichrysum* species are traditionally used to treat inflammatory skin disorders and improve wound healing (Grčić et al., 2017). Other species in the Asteraceae family have also been traditionally reported to have this activity and is attributed to their ability to enhance keratinocyte proliferation and the remodeling of the skin extracellular matrix (Carvalho Jr. et al., 2018). There were obvious adverse effects on cell viability at 200 and 400 µg/ml. However, at concentrations between 3.125 and 25 µg/ml cell proliferation seems to be induced as compared to the negative control. The effects of HO-MeOH on human keratinocyte proliferation is, therefore, concentration sensitive. The anti-proliferative effect of HO-MeOH was determined in murine macrophages in order to obtain non-lethal doses of extract that could then be used to assess the nitric oxide inhibitory effects of the extract as well as the inhibitory effects on the expression of specific genes associated with inflammation. The cell viability was normalized against the negative DMSO vehicle control (0.625% v/v). The IC_50_ of HO-MeOH was 61.29 ± 10.51 µg/ml on RAW264.7 cells. The toxic inducer of DMSO (5% v/v) completely inhibited cell proliferation (data not shown) (**Figures 2B** and **S9B**).

### Interleukin-1α Inhibition

**Figure 3:**
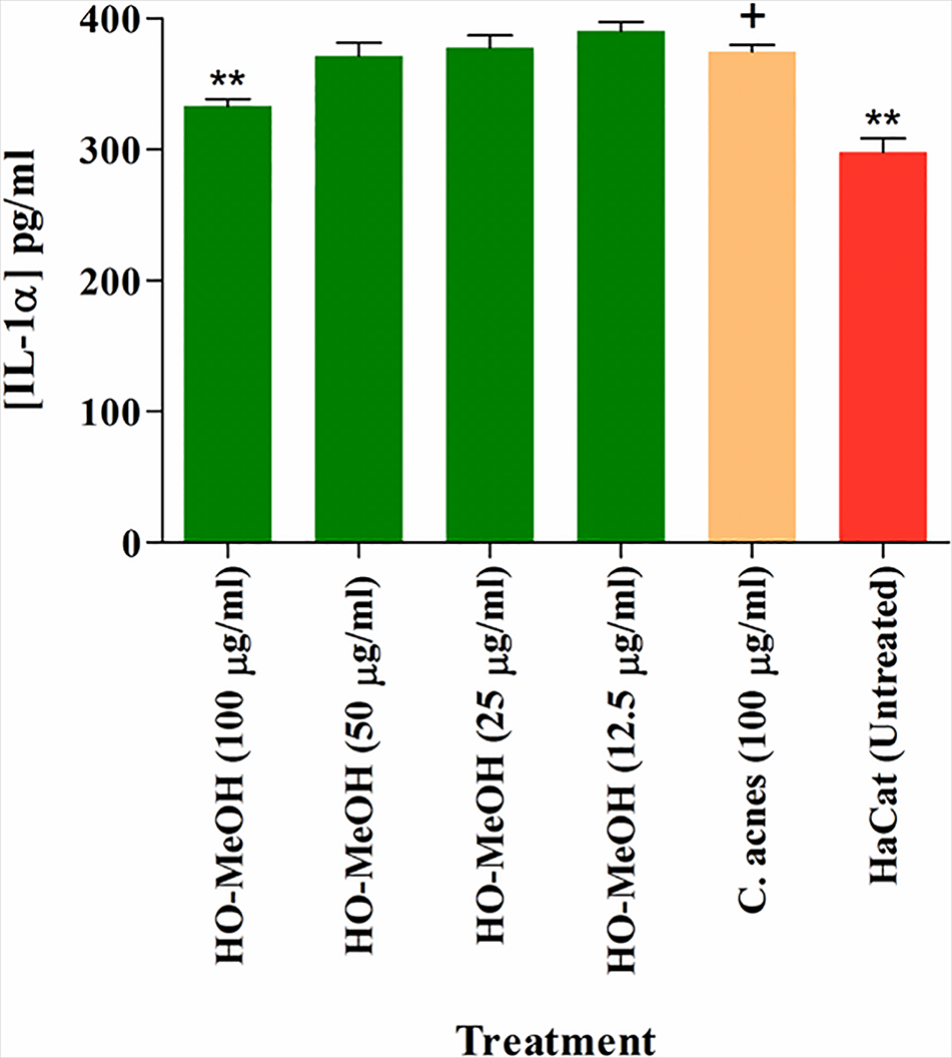
Inhibition of interleukin-1α (IL-1α) protein levels in *Cutibacterium* acnes-induced human keratinocytes treated with the *Helichrysum odoratissimum* methanol extract. **p < 0.01. The “+” indicates the control column to which all the data sets were compared using the Dunnet’s multiple comparison test.

The LPS-induced cytokine production was used as a control for IL-1α production. The effects of HO-MeOH, together with *C. acnes* stimulation, was used to assess the anti-inflammatory activity of HO-MeOH in response to *C. acnes*-induced cytokine production specifically. The induction of HaCaT cells using heat-killed *C. acnes* showed a significant difference when compared to the untreated HaCaT cell control. The *C. acnes* caused an increase in IL-1α from a basal concentration of 298.10 ± 20.88 pg/ml to 374.69 ± 10.17 pg/ml. A significant difference was also observed between the *C. acnes* treated cells and 100 µg/ml HO-MeOH, which reduced IL-1α protein levels to 333.18 ± 10.92 pg/ml (**Figure 3**).

### Lipase Inhibition

**Figure 4:**
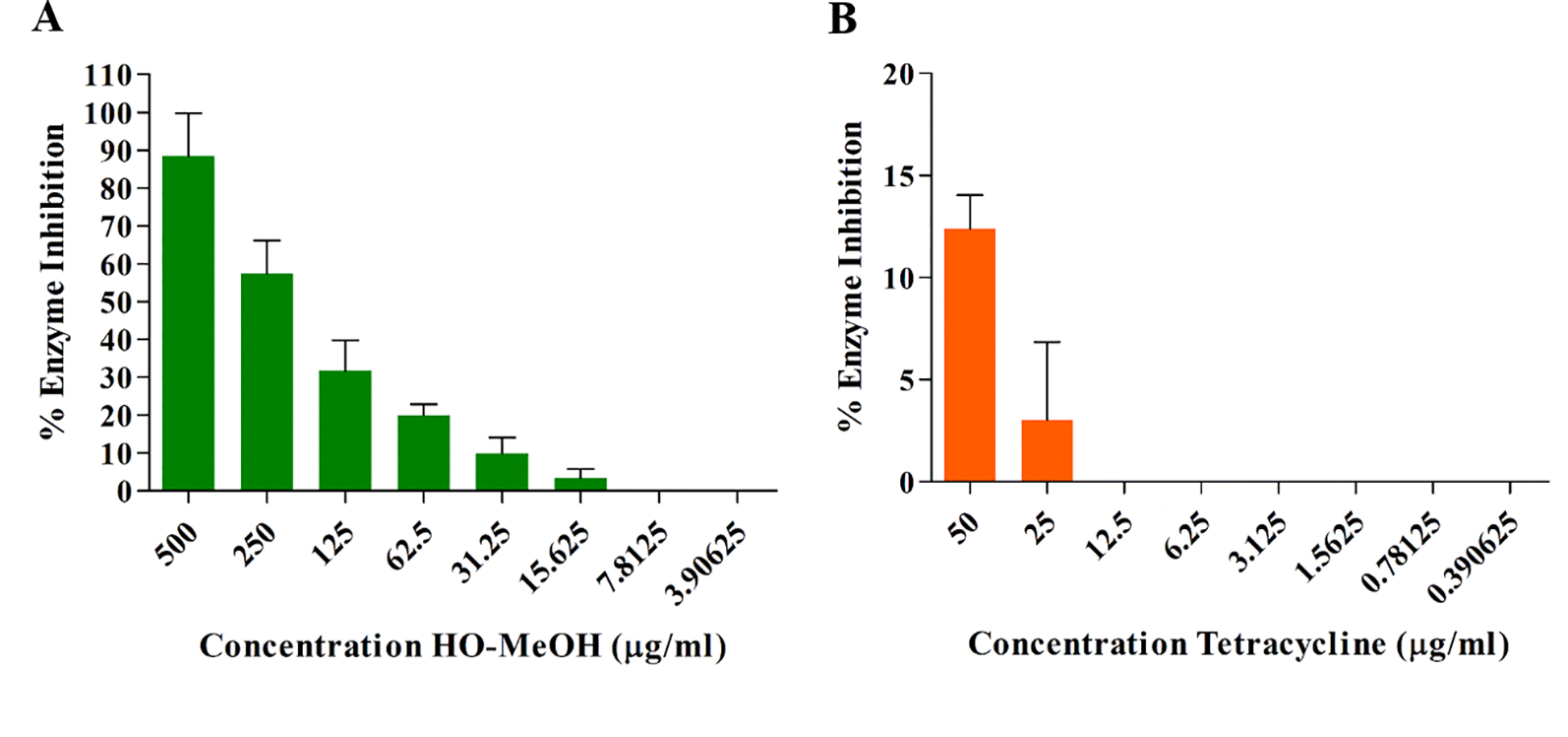
Inhibition of *Candida rugosa* lipase activity by the *Helichrysum odoratissimum* methanol extract **(A)** and tetracycline **(B)**.

The HO-MeOH extract exhibited an IC_50_ of 157.50 ± 6.85 µg/ml against lipase activity (**Figures 4A** and **S10**). Tetracycline showed low levels of lipase inhibition even at the highest concentration tested (**Figure 4B**). Previous studies have shown the ability of *C. acnes* lipase enzyme to use 2, 3-dimercapto-1-propanol tributyrate as a substrate, which suggests that the results obtained using lipase from *C. rugosa* would provide relatable inhibitory data for *C. acnes* lipase inhibition (Batubara et al., 2014). Previous reports of the activity of tetracyclines on lipase inhibition show similar results observed in this study. Tetracycline hydrochloride, chlortetracycline hydrochloride, and demeclocycline hydrochloride showed 76, 76, and 80% inhibition at 500 µg/ml, respectively. At 50 µg/ml, chlortetracycline hydrochloride and tetracycline hydrochloride showed 18 and 20% inhibition, respectively. Concentrations as high as 0.008 M (3.56 mg/ml) have only shown 89% inhibition (Hassing, 1971; Puhvel and Reisner, 1972). More recent studies have demonstrated similar results for tetracycline as a lipase inhibitor with IC_50_ as high as 471.3 ± 5.5 µg/ml (Batubara et al., 2009). This study also tested the methanol extracts of several Indonesian plant species traditionally used for skin eruptions or acne and showed similar results. The methanol extracts of *Caesalpinia sappan*, *Curcuma longa*, *Curcuma xanthorrhiza*, *Goniothalamus macrophyllus*, and *Lepisanthes amoena* exhibited IC_50_ of 150.0 ± 1.0, 80.1 ± 3.9, 274.5 ± 5.7, 120.0 ± 5.3, and 151.7 ± 3.5 µg/ml, respectively, against *C. acnes* lipase. The use of HO-MeOH would, therefore, have a larger effect on prevention of sebum degradation by *C. acnes*, possibly preventing the formation of the glycerol nutrient source and release of free fatty acids, in turn, preventing *C. acnes* proliferation in the pilosebaceous unit and reducing inflammation caused by irritant free fatty acids.

### Anti-Inflammatory Activity

#### Inhibition of Pro-Inflammatory Cytokines

**Figure 5:**
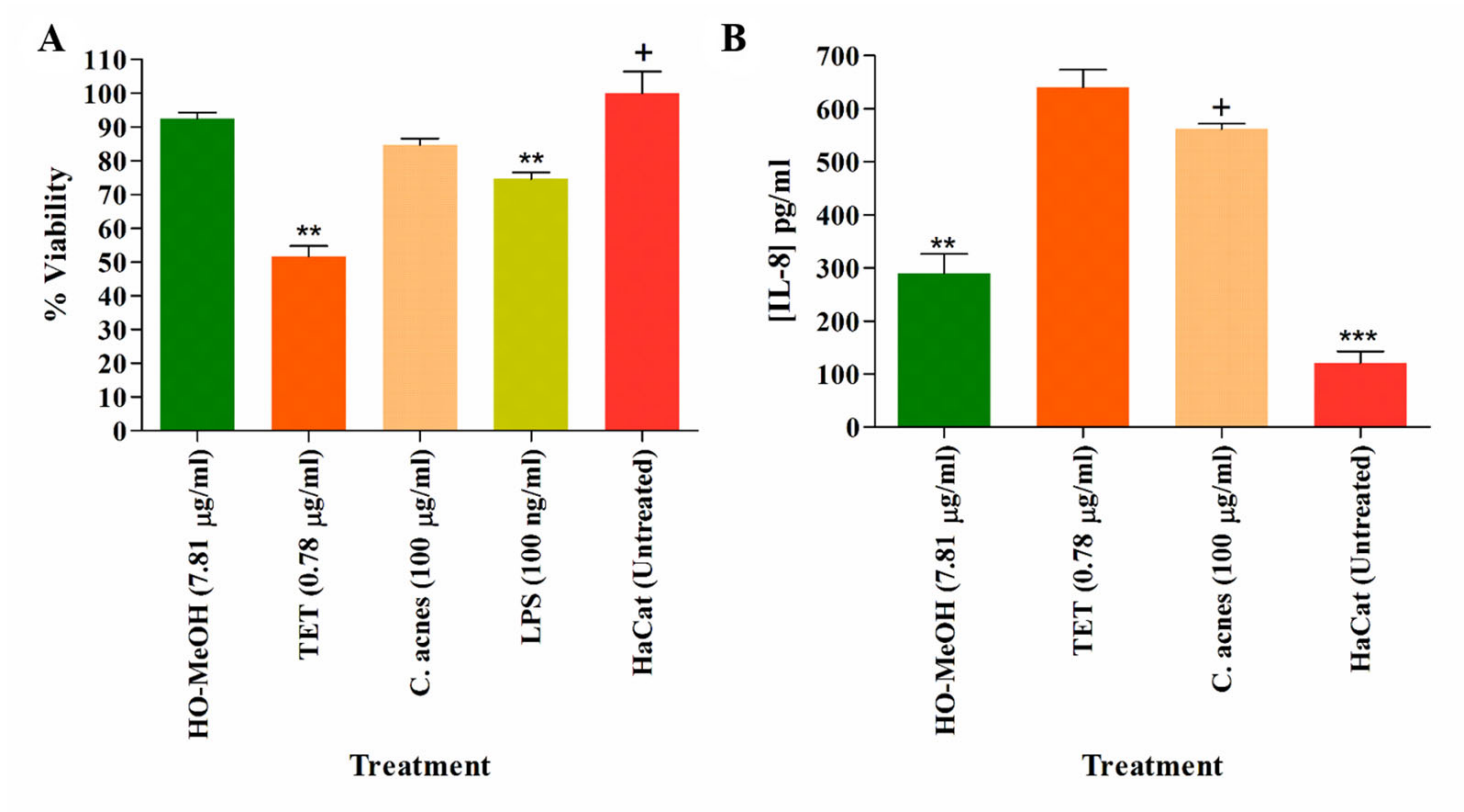
The cell viability of human keratinocytes (HaCaT) **(A)**, inhibition of pro-inflammatory cytokine IL-8 **(B).** **p < 0.01; ***p < 0.001. The “+” indicates the control column to which all the data sets were compared using the Dunnet’s multiple comparison test.

The effects of LPS and heat-killed *C. acnes* induction were assessed using several inflammatory marker cytokines including IL-1β, IL-6, IL-8, IL-10, IL-12p70, and TNF-α. The induction of HaCaT cells using LPS was used as a control for cytokine production. The effects of HO-MeOH, together with *C. acnes* stimulation, was used to assess the anti-inflammatory activity of HO-MeOH in response to *C. acnes*-induced cytokine production specifically. The protein levels of IL-6 and IL-8 were found to be induced within detectable limits and were reported. The HaCaT cell viability observed for the treatment of cells was greater than 70%, except in keratinocytes treated with tetracycline, where 51.61% viability was observed (**Figure 5A**). This, therefore, suggests that any observed inhibition of IL-6 or IL-8 was not due to cytotoxicity against HaCaT cells for HO-MeOH (Wang et al., 2014). *C. acnes*-induced HaCaT cells resulted in significant increases of IL-8 when compared to the untreated cell control, which was not true for IL-6, even though levels of IL-6 were increased when compared to the untreated cell control (**Figures 5B** and **S6**). The HO-MeOH, tested at the MIC (7.81 µg/ml) exhibited 48.31% reduction in IL-8 protein levels from 561.58 ± 14.85 to 290.24 ± 21.02 pg/ml (**Figure 5B**). Tetracycline, in both cases had a stimulatory effect on IL-6 and IL-8 levels.

#### Inhibition of Cyclooxygenase-II Activity

**Figure 6:**
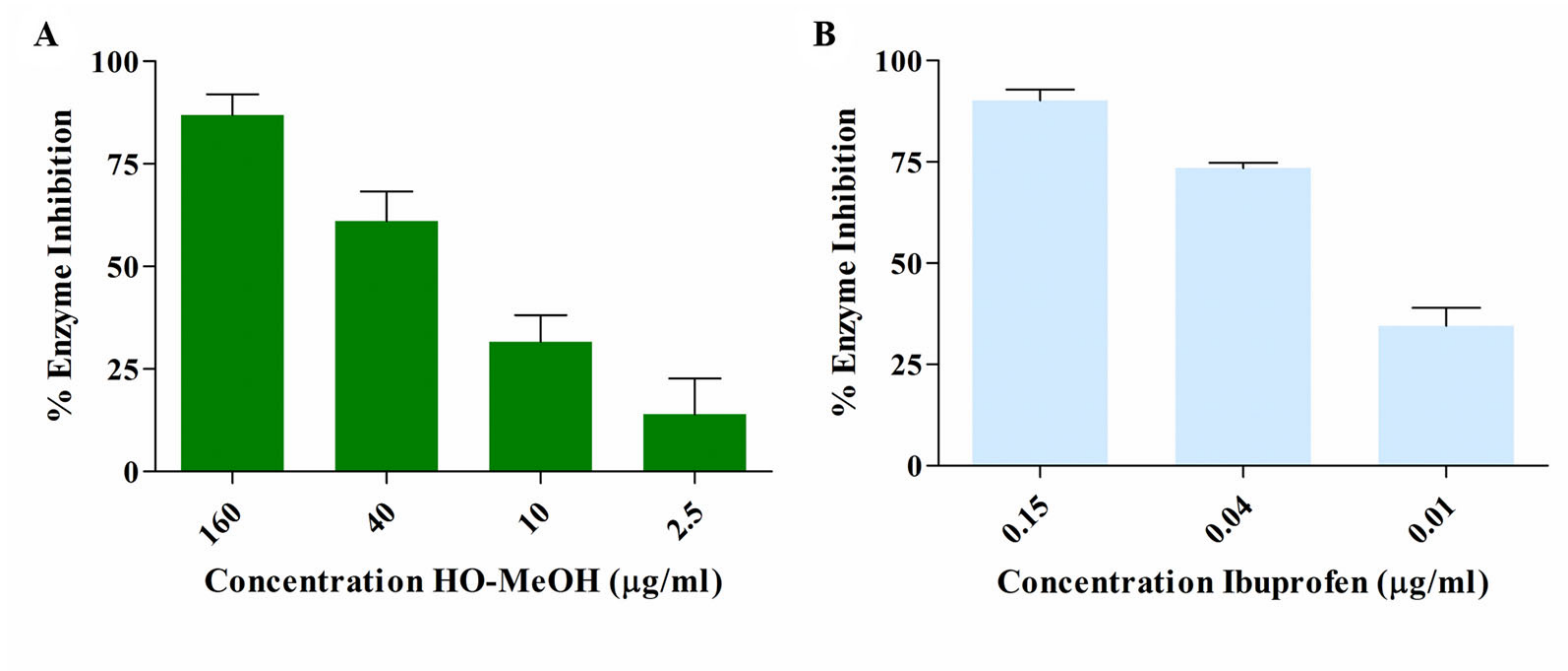
Dose-dependent enzyme inhibition of cyclooxygenase II enzyme activity by the *Helichrysum odoratissimum* methanol extract **(A)** and ibuprofen **(B)**.

The COX-II inhibitory activity of HO-MeOH exhibited a dose-response with an IC_50_ of 22.87 ± 6.48 µg/ml (**Figures 6A** and **S11A**). This was compared with the inhibition of Ibuprofen, a known inhibitor of COX-II activity with an IC_50_ of 0.75 ± 0.01 µM (0.15 µg/ml) (**Figures 6B** and **S11B**). The GC-MS profile of the extract identified linoleic acid as one of the major constituents in the extract. This fatty acid has been previously reported to have COX inhibitory activity, with a higher selectivity toward COX-II. The reported IC_50_ values for linoleic acid were 94 µM (26.17 µg/ml) and 170 µM (43.33 µg/ml) for COX-II and COX-I, respectively (Ringbom et al., 2001). The HO-MeOH showed similar results to the ethanol extract of *Helichrysum kraussii,* which showed 57.15 ± 8.00% inhibition at a concentration of 10 µg/ml. The positive control Ibuprofen, at the same concentration, inhibited 90.17 ± 3.12% of COX-II activity (Twilley et al., 2017b). Ibuprofen in the current study, exhibited similar COX-II inhibition, with 90.09% observed at 2.06 µg/ml (10 µM). The activity of arzanol, a prenylated phloroglucinol *α*-pyrone compound from *H. italicum* has been identified as an inhibitor of PGE_2_ production. The presence of these types of compounds has been identified in *H. odoratissimum* and could, therefore, be responsible for the observed COX-II activity (Lourens et al., 2008; Guinoiseau et al., 2013). The presence of benzothiazole was identified as one of the major constituents in the extract using GC-MS. Heterocyclic compounds, including those with the benzothiazole skeleton, have a vast range of pharmaceutical and biological activities, including anti-inflammatory activity. The presence of this compound and its derivatives could potentially contribute to the COX-II inhibitory activity (Yatam et al., 2018).

#### Nitric Oxide Scavenging Activity and Intracellular Nitric Oxide Inhibition

The HO-MeOH extract exhibited NO with an IC_50_ of 214.90 ± 13.27 µg/ml. The positive control vitamin C had an IC_50_ of 35.24 ± 7.85 µg/ml (**Table 3**). The 2,2-diphenyl-1-picrylhydrazyl (DPPH) scavenging activity of the methanolic extract of *H. odoratissimum* (incorrectly identified as *Helichrysum dasyanthum* by Lourens et al., 2004) exhibited potent anti-oxidant activity with an IC_50_ of 12.33 µg/ml (Lourens et al., 2008), indicating its ability to scavenge free radicals. Several *Helichrysum* species have been investigated for their ability to scavenge free radicals using the DPPH model (Lourens et al., 2004; Albayrak et al., 2010). The nitric oxide scavenging activity of other *Helichrysum* species have also been tested. The aqueous leaf extract of *Helichrysum pedunculatum* exhibited 68% inhibition of NO radicals at 800 µg/ml. The aqueous extract of *Helichrysum longifolium* showed 31.71, 42.00, 62.28, and 64.96% inhibition at 200, 400, 600, and 800 µg/ml, respectively (Aiyegoro and Okoh, 2010). This data suggests that HO-MeOH is a more potent inhibitor of NO when compared to some other species of *Helichrysum* and was further investigated for its effects against intracellular NO production (Aiyegoro and Okoh, 2009).

**Figure 7:**
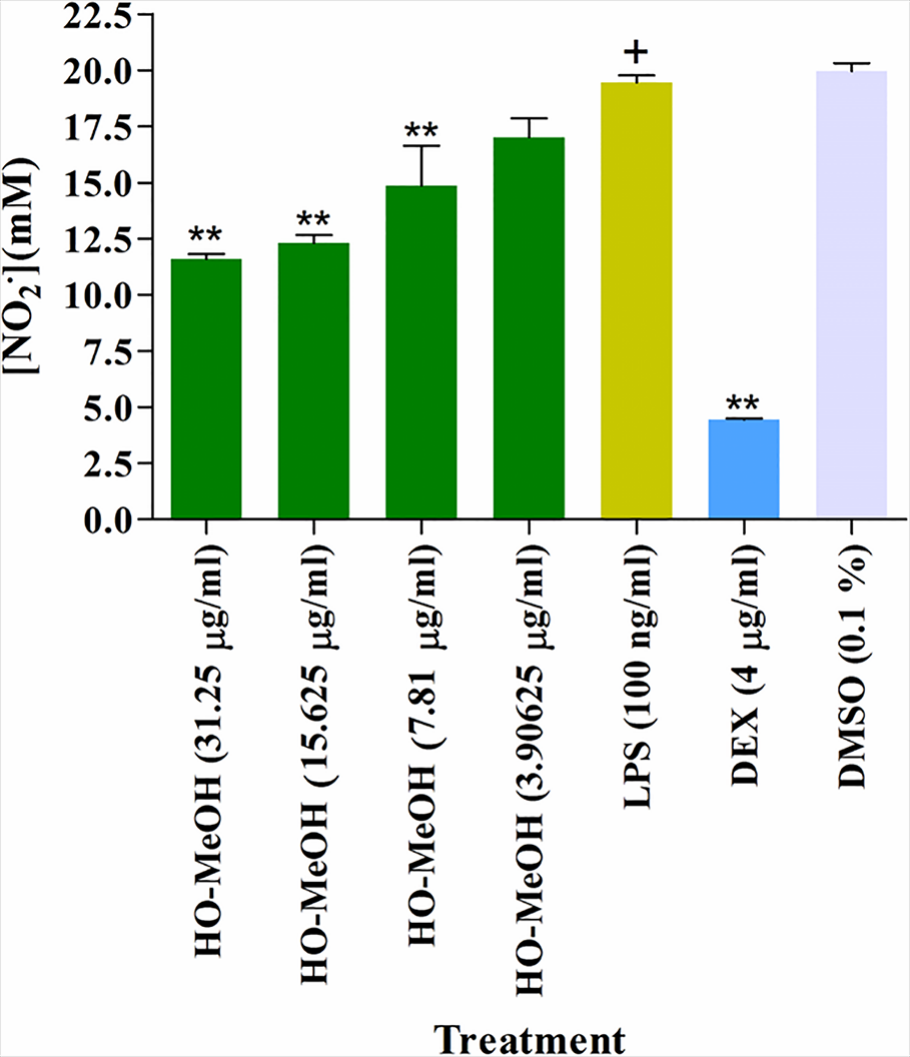
Inhibition of cellular nitric oxide in murine macrophage cells (RAW264.7) induced using lipopolysaccharide. **p < 0.01.

The inhibition of cellular NO was tested using murine macrophages (RAW264.7) where induction of NO was achieved with treatment of the cells with LPS. The induction of NO by LPS is said to occur *via* activation of inducible iNOS and is quantitatively inducible in several cell types, including macrophages. The NO produced through the iNOS pathway are associated with many physiological processes of disease and inflammation and is, therefore, an important target, considering that acne is a chronic inflammatory disorder (Lin et al., 2003). The inhibition of NO was determined through the concentration of nitrite (NO_2_^−^). Inhibition of NO was compared with LPS-induced RAW264.7 cells. The HO-MeOH extract showed significant inhibition of NO at 7.81, 15.625, and 31.25 µg/ml. The concentration of nitrite was reduced from 19.46 µM in the LPS-stimulated cells to 14.86, 12.31, and 11.60 µM, respectively. The positive control, dexamethasone showed significant inhibition of NO at 4 µg/ml (10 µM). The negative control 0.1% DMSO showed no inhibitory effect on NO production, indicating that the observed inhibition was likely due to pre-incubation with HO-MeOH. The intracellular NO scavenging activity showed a reduction of 40.39 and 36.74% reduction in NO production levels at 31.25 and 15.625 µg/ml, respectively (**Figure 7**). The NO scavenging activity of HO-MeOH was similar for both these concentrations and correlated well with the intracellular inhibitory activity. Previous reports on the radical scavenging activity include potent DPPH scavenging activity of the ethanol extract with an IC_50_ of 5.13 µg/ml (Twilley et al., 2017a). A study by Mao et al. (2017) highlighted the anti-inflammatory activity of flavonoids from *Helichrysum arenarium* in the prevention of inflammation-induced atherosclerosis. Flavonoids isolated from the methanolic flower extract of *H. odoratissimum* include 3,5-dihydroxy-6,7,8-trimethoxyflavone, 3-*O*-methylquercetin, and 4,2’,4’-trihydroxy-6’-methoxychalcone (helichrysetin) (Van Puyvelde et al., 1989; Lourens et al., 2008). These constituents could potentially explain the anti-inflammatory activity of the HO-MeOH extract used in this study. The results warrant the future investigation of flavonoid-enriched fractions of *H. odoratissimum* for anti-inflammatory activity. The NO scavenging activity and intracellular NO inhibitory activity justified further investigation into possible mechanisms of this anti-inflammatory activity.

#### Inhibition of Inflammatory Gene Expression

**Figure 8:**
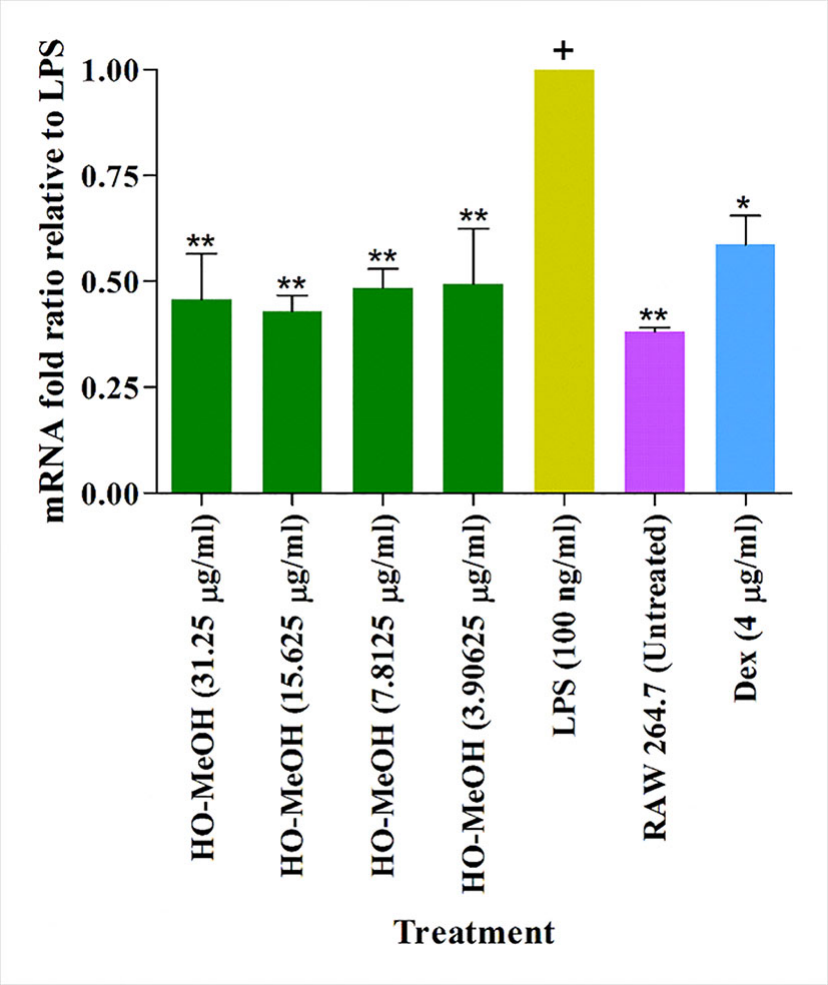
Inhibition of inducible nitric oxide synthase (iNOS) gene expression in murine macrophage cells (RAW264.7). *p < 0.05; **p < 0.01. The “+” indicates the control column to which all the data sets were compared using the Dunnet’s multiple comparison test.

The use of murine macrophages (RAW264.7) in the investigation of anti-inflammatory activity of natural compounds is common practice. These cells behave much like human macrophages, with the induction of LPS resulting in the activation of signal pathways and gene expression. The overexpression of both cyclooxygenase-II and inducible iNOS resulting in the formation of prostaglandin-E_2_ (PGE_2_) and NO are among the most prominent effects of LPS induction (Ma et al., 2018). The gene expression of several inflammatory genes related to acne, including COX-II and iNOS, were investigated in LPS-induced RAW264.7 cells treated with HO-MeOH. The *β*-actin gene was used as the reference (housekeeping) gene. The gene expression levels of treated cells were normalized relative to the gene expression of *β*-actin. Cells induced with LPS only were set to 1 as the maximum level of overexpression. Therefore, values <1 indicated inhibition of gene expression and values >1 indicated gene overexpression relative to LPS stimulation. The melting curves and amplification plot indicated that there was no formation of primer-dimers with the PCR reaction. In an attempt to correlate gene expression data to the anti-inflammatory bioassays, only the COX-II and iNOS gene expression were reported. The HO-MeOH exhibited inhibition of COX-II expression. However, there was no significant difference between the COX-II expression levels in the HO-MeOH treatments when compared to those observed in LPS-induced expression. The positive control, dexamethasone exhibited a significant reduction in COX-II expression with a 2.63 reduction in fold change from 1 to 0.38 (**Figure S7**). On the other hand, the inhibition of NO observed in LPS-induced RAW264.7 cells treated with HO-MeOH is most likely due to inhibitory gene expression of iNOS gene expression. The HO-MeOH extract showed significant reduction of iNOS expression at all concentrations with relatively consistent inhibition over the tested range (**Figure 8**). Chu et al. (2009) observed that the extracts of three Chinese herbs *Drynaria baronii*, *Angelica sinensis*, and *Cornus officinalis* had a greater inhibitory effect on the gene expression of iNOS as compared to that of COX-II. They also demonstrated that PGE_2_ was inhibited to a lesser degree than that of NO, which correlated well with the results observed in the COX-II enzyme inhibition assay and the cellular NO inhibition assay. The HO-MeOH inhibited COX-II activity and consequently, the PGE_2_ levels by 50% at a concentration of 22.87 µg/ml while 31.25 µg/ml caused a 59% reduction in NO. The results indicated that the HO-MeOH extract inhibited the functional COX-II enzyme, as opposed to the gene expression of this acute inflammatory biomarker. The pathogenesis of *C. acnes* should be a core target when developing acne therapies from natural sources, as the inflammatory cascade can often result in scarring of the skin.

### Hyaluronidase Inhibition

**Figure 9:**
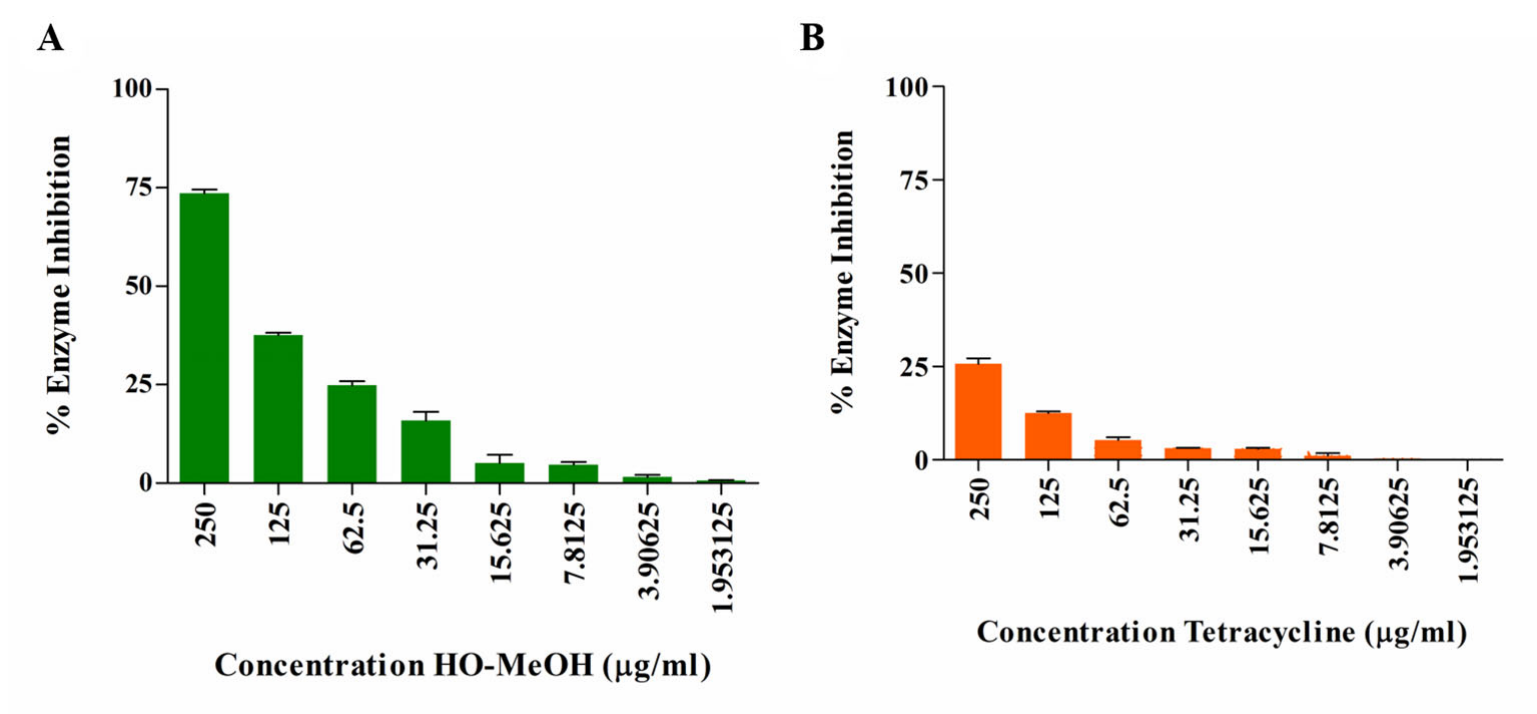
Inhibition of *Streptomyces pyogenes* hyaluronidase activity by *Helichrysum odoratissimum* methanol extract **(A)** and tetracycline **(B)**.

Dose-dependent inhibition of HYAL activity was observed for HO-MeOH with an IC_50_ of 145.45 ± 6.22 µg/ml (**Figures 9A** and **S12**). Tetracycline showed a dose-response inhibition of HYAL, with reduced inhibitory activity being observed with an IC_50_ > 250 µg/ml (**Figure 9B**). There are not many reports on the inhibition of bacterial hyaluronidases, as many studies report on the inhibitors of bovine testes HYAL. Triterpenoid compounds glycyrrhizin and glycyrrhetinic acid isolated from the root of *Glycyrrhiza glabra* have shown inhibition of *Streptococcus agalactiae* with IC_50_ of 0.44 mM (362 µg/ml) and 0.06 mM (28 µg/ml), respectively. Inhibition was also observed against *Streptococcus equisimilis* HYAL activity with IC_50_ of 1.02 mM (839 µg/ml) and 0.26 mM (122 µg/ml). Flavonoids rutin and silybin were also identified as inhibitors of *S. agalactiae* HYAL activity. The inhibitory activity of HO-MeOH, therefore, compares well with previously published data on bacterial hyaluronidase inhibitors (Hertel et al., 2006). Considering that flavonoids and diterpenes have previously been isolated from *H. odoratissimum* it is not surprising that HO-MeOH showed inhibition of this enzyme. The use of *Streptococcus pyogenes* HYAL as a model for predicting the inhibitory effects of HO-MeOH on *C. acnes* HYAL is advantageous, as these HYALs share homology when comparing amino acid sequences. The HYAL type identified in several *C. acnes* is strain specific. Phylotypes of *C. acnes* belonging to type I_A_ result in the production of larger fragments of HA whereas, those belonging to the type I_B_ and II produce smaller fragments of more consistent sizes. Considering that HA fragments produced *via* HYAL activity are used as a nutritive source for *C. acnes* and can reduce the immune response during bacterial colonization, inhibitors of this enzyme should be considered advantageous in acne therapy (Nazipi et al., 2017). There are currently no identified acne therapies focused on hyaluronidase inhibition, which warrants further investigation into the isolation of constituents from HO-MeOH as hyaluronidase inhibitors.

## Conclusion

In conclusion, this study provides scientific validation for the traditional use of *H. odoratissimum* as a possible treatment for acne, based on direct antimicrobial effects as well as the inhibition of key targets in the pathogenic processes associated with the opportunistic pathogen, *C. acnes* in the progression of this skin disorder. The study identified the potent antimicrobial activity of the methanolic extract of *H. odoratissimum* against *C. acnes* and the anti-biofilm activity of this extract particularly affecting the initial step of bacterial adhesion. The study also identified the anti-inflammatory potential of *H. odoratissimum* inhibiting inflammatory cytokine IL-8, COX-II, and NO. The mechanism of action for NO inhibition was through the inhibition if iNOS gene expression. The hyaluronidase inhibitory activity of *H. odoratissimum* indicated a potential source of bacterial hyaluronidase inhibitors, a key enzyme involved in bacterial spread and tissue injury, not only in *Cutibacterium* species but also other *Streptococcus* species. *C. acnes* has also been identified as a pathogen involved in joint infections in patients implanted with prosthetic medical devices. The anti-adhesion activity of the extract can perhaps be employed as a coating for medical devices prior to insertion for the prevention of joint inflammation.

## Data Availability Statement

The datasets generated for this study are available on request to the corresponding author.

## Author Contributions

MNDC conducted the study and compiled the manuscript. SK hosted MC in his laboratory and provided guidance with the RAW264.7 cell culture, MTT antiproliferative activity, intracellular NO, and gene expression work. LL provided guidance for the COX-II enzyme inhibition study. NL acted as the supervisor for the study and also provided a critical assessment and editorial guidance on the manuscript.

## Funding

The authors would like to thank the South African Research Chairs Initiative and the Indigenous Knowledge Systems Framework through the National Research Foundation (NRF) (Grant ID: 98334 & 105169) for funding the research.

## Conflict of Interest

The authors declare that the research was conducted in the absence of any commercial or financial relationships that could be construed as a potential conflict of interest.
