# Supplementary material for: Exploring the Anti-Acne Potential of Impepho [Helichrysum odoratissimum (L.) Sweet] to Combat Cutibacterium acnes Virulence
Source: Front Pharmacol. 2020 Jan 30;10:1559. doi: 10.3389/fphar.2019.01559 (PMC7002546; doi:10.3389/fphar.2019.01559)
Supplement: Supplementary file 1 [file DataSheet_1.docx]

**Supplementary Data:**


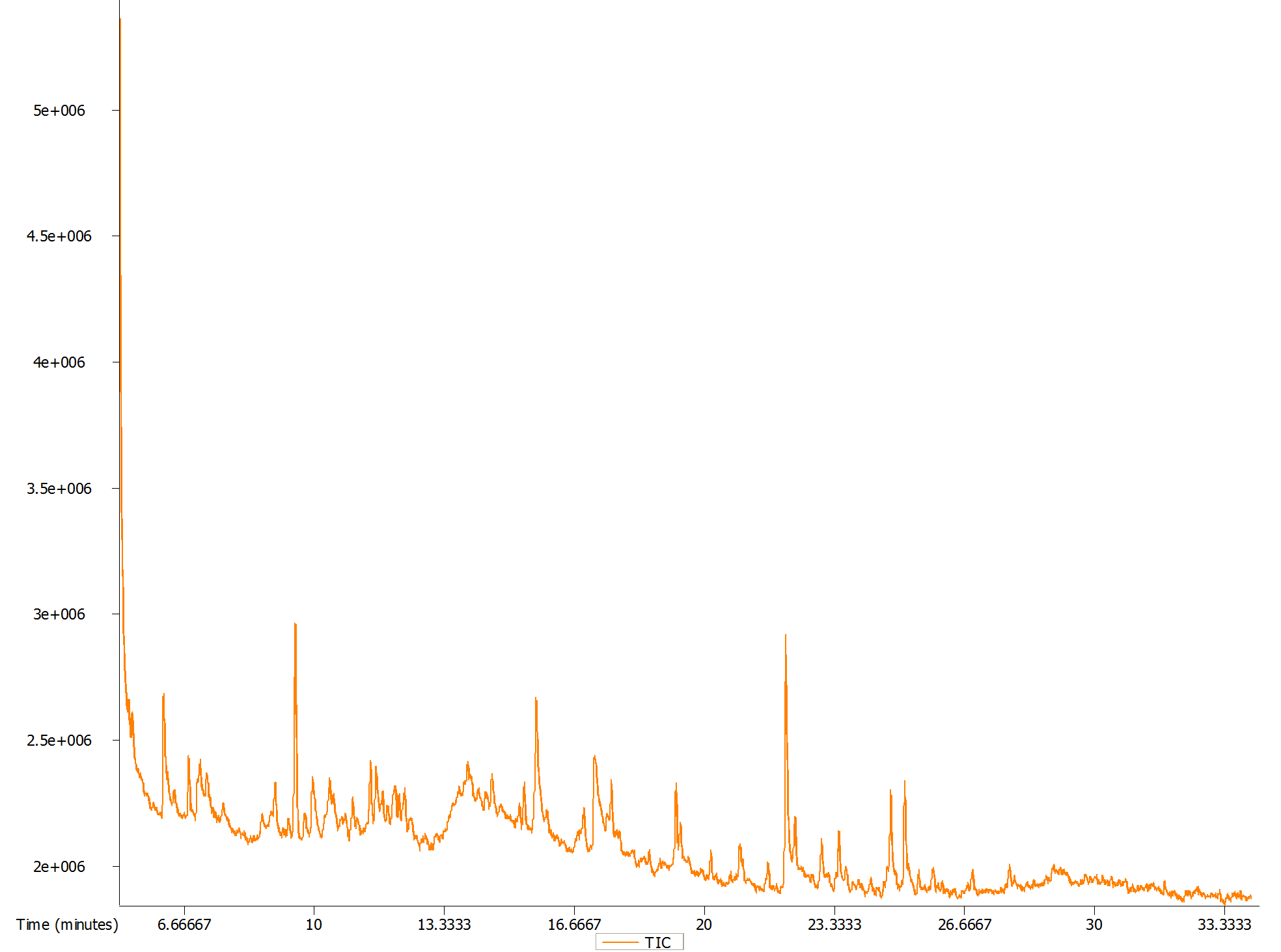


Figure S1: GC-MS chromatogram of the methanolic extract of *H. odoratissimum*


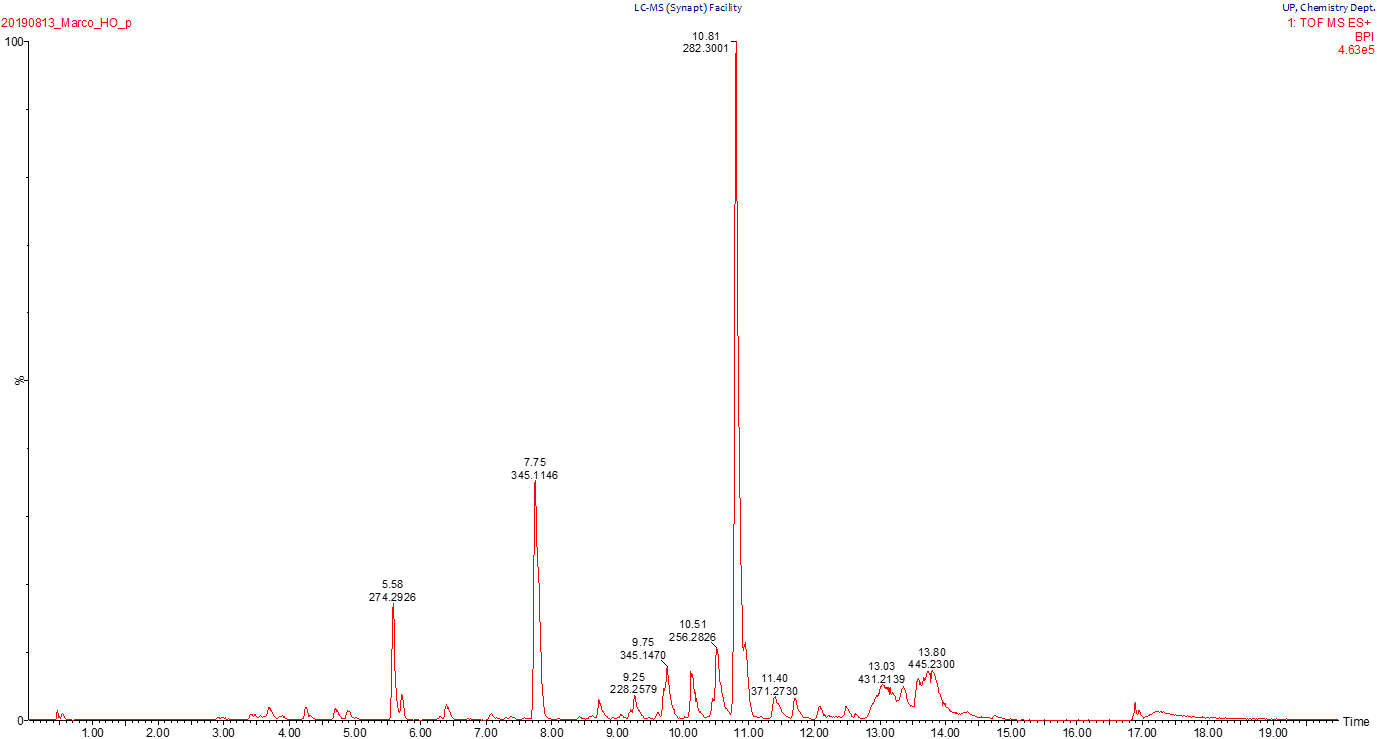


Figure S2: Positive ionisation LC-MS chromatogram of methanolic extract of *H. odoratissimum*


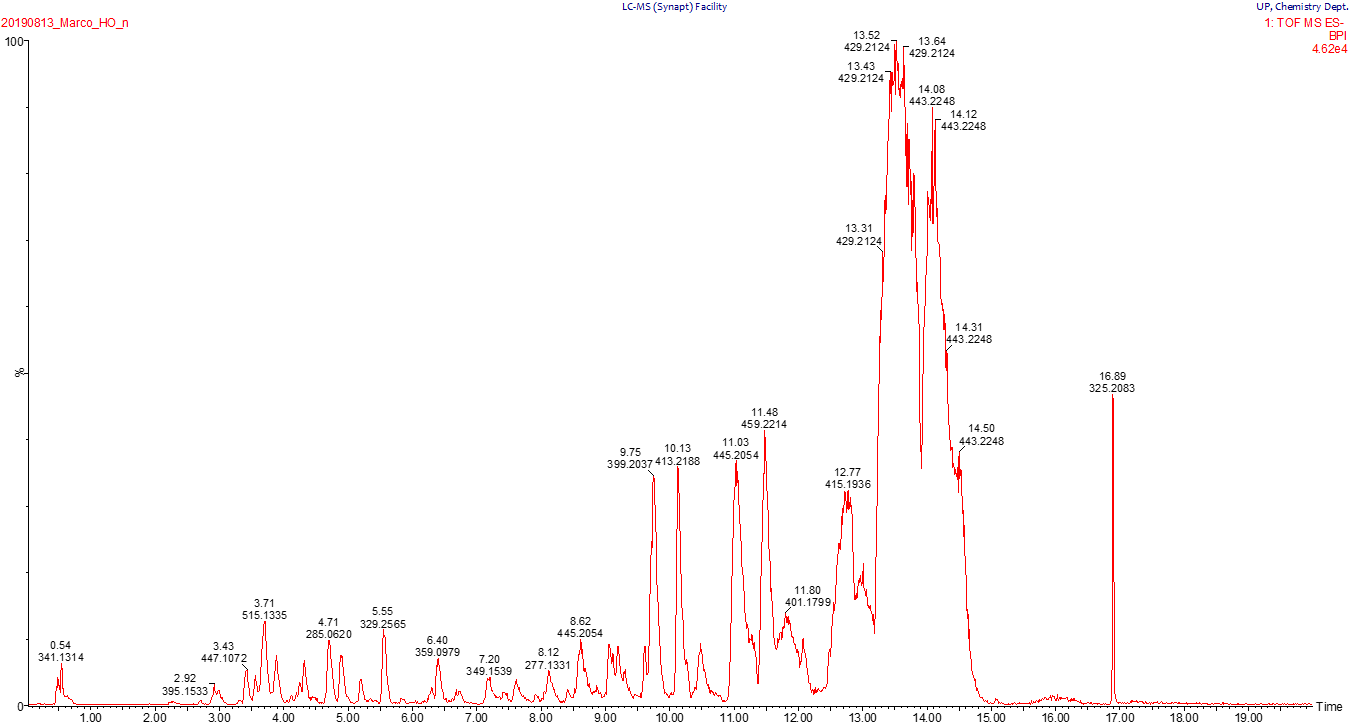


Figure S3: Negative ionisation LC-MS chromatogram of methanolic extract of *H. odoratissimum*

Figure S4: Mass fragmentation pattern for peak at 3.71 minutes (red) and corrected mass fragmentation pattern using lock mass correction (black)


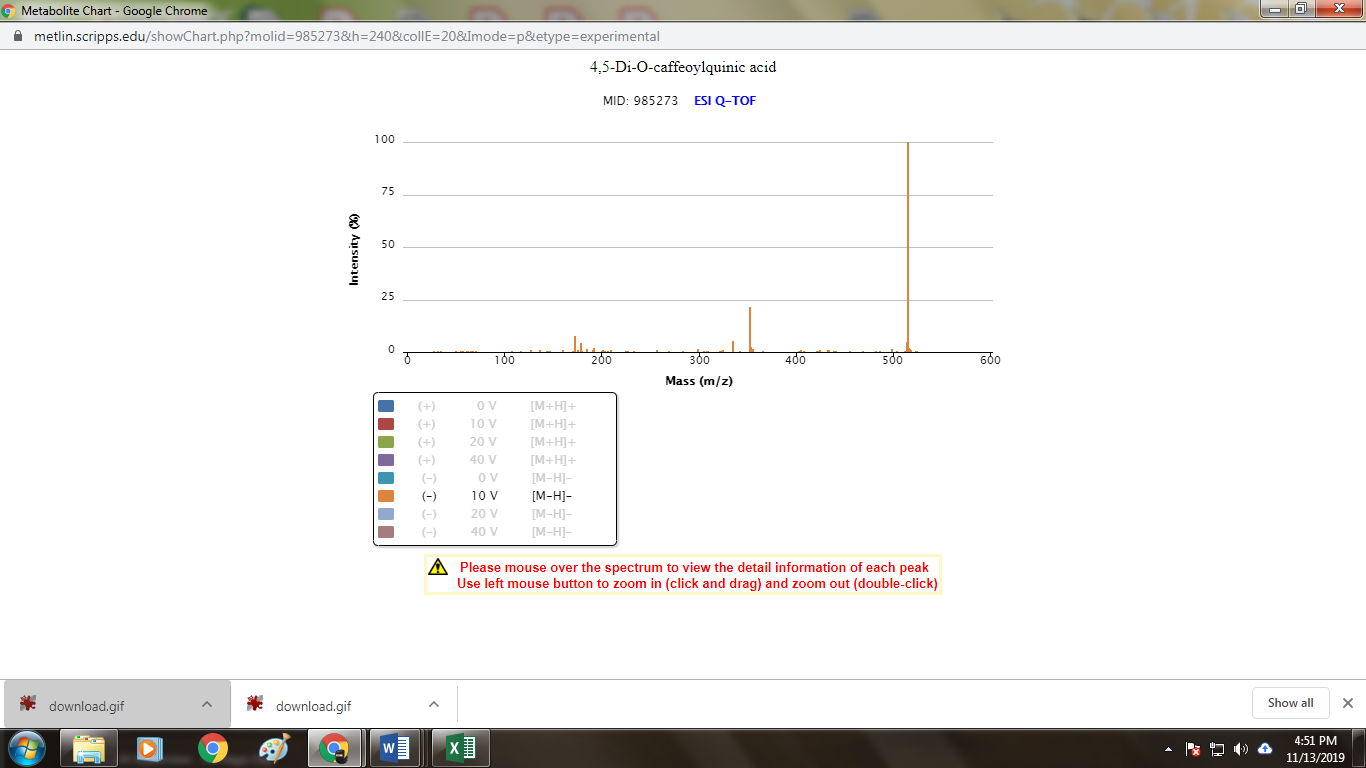


Figure S5: Mass fragmentation pattern for 4, 5-Di-O-caffeoylquinic acid from METLIN database





Figure S6: Inhibition of pro-inflammatory cytokine IL-6





Figure S7: Inhibition of cyclooxygenase II (COX-II) gene expression in murine macrophages (RAW264.7)


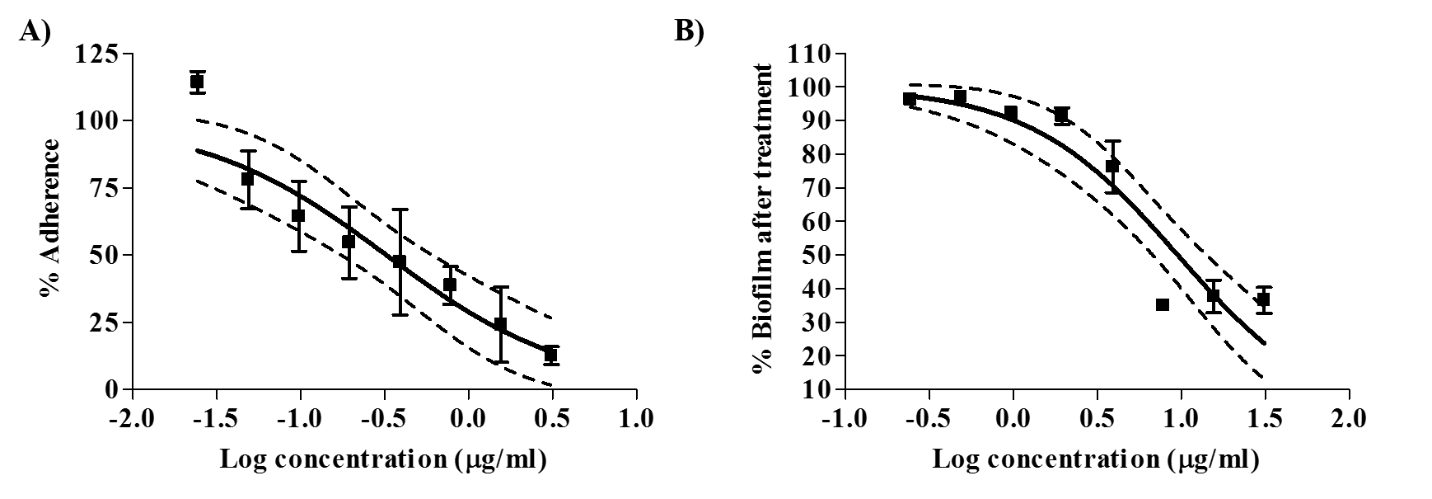


Figure S8: Anti-adhesion dose-response curve of **A)** tetracycline and **B)** the biofilm eradication dose-response curve of the methanolic extract of *H. odoratissimum*


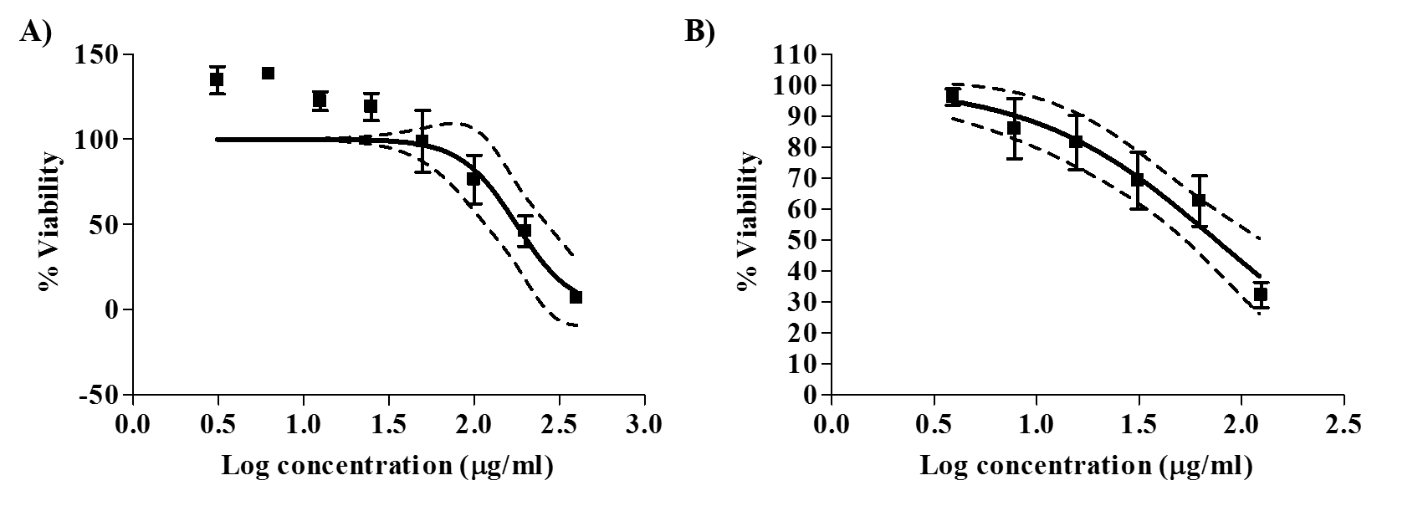


Figure S9: Effects of the methanolic extract of *H. odoratissimum* on **A)** human keratinocyte (HaCaT) and **B)** murine macrophage (RAW264.7) viability


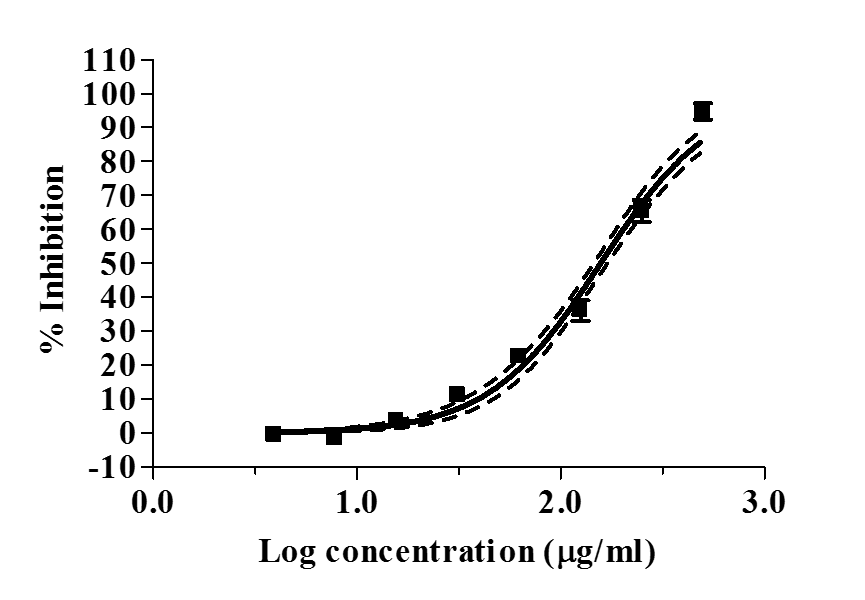


Figure S10: Dose-response curve of lipase inhibition by the methanolic extract of *H. odoratissimum*


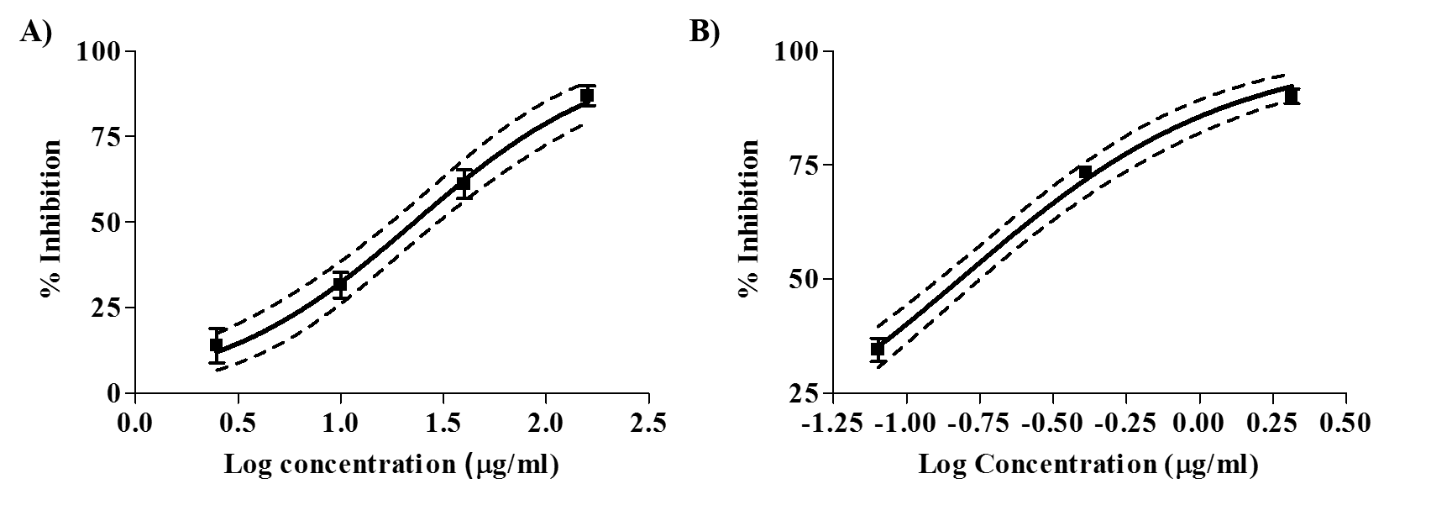


Figure S11: Dose-response curves for the cyclooxygenase I inhibition by the methanolic extract of **A)** *H. odoratissimum* and **B)** ibuprofen


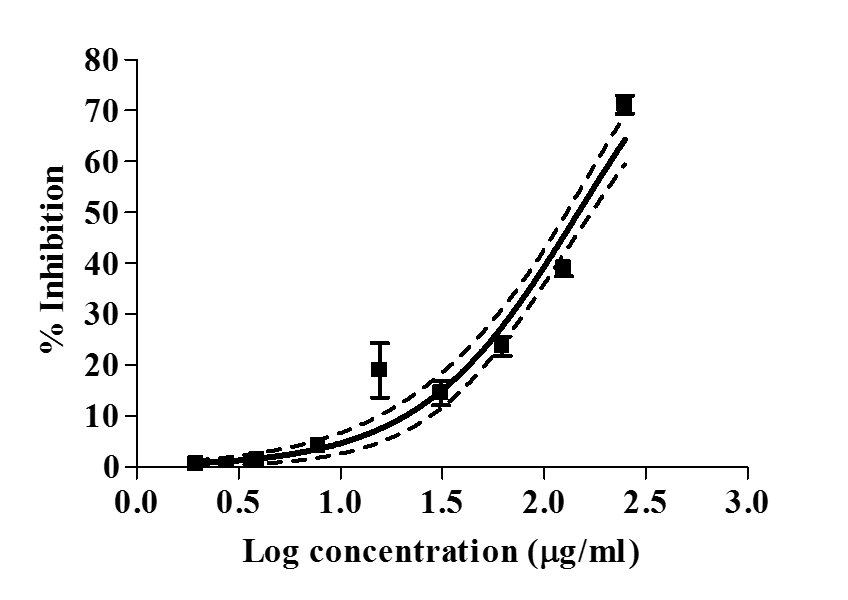


Figure S12: Dose-response curve of hyaluronidase inhibition by the methanolic extract of *H. odoratissimum*
